# Screening and analysis of programmed cell death related genes and targeted drugs in sepsis

**DOI:** 10.1186/s41065-025-00403-w

**Published:** 2025-03-19

**Authors:** Juanjuan Song, Kairui Ren, Yi Wang, Dexin Zhang, Lin Sun, Zhiqiang Tang, Lili Zhang, Ying Deng

**Affiliations:** 1 Department of Emergency, The Second Affiliated Hospital of Harbin Medical University, No.148 Baojian Road, Nangang District, Harbin, 150086 Heilongjiang China; 2 Department of Emergency, Peking Union Medical College Hospital, Chinese Academy of Medical Science, Beijing, 100730 China; 3 Department of Critical Care Medicine, The Second Affiliated Hospital of Harbin Medical University, Harbin, 150086 China; 4 Department of Respiratory and Critical Care Medicine, The Second Affiliated Hospital of Harbin Medical University, Harbin, 150086 China

**Keywords:** Sepsis, Multiple organ dysfunction, Programmed cell death, Bioinformatics, Mendelian randomization analysis

## Abstract

**Objective:**

This study employed bioinformatics techniques to identify diagnostic genes associated with programmed cell death (PCD) and to explore potential therapeutic agents for the treatment of sepsis.

**Methods:**

Gene expression profiles from sepsis patients were analyzed to identify differentially expressed genes (DEGs) and hub genes through Weighted Gene Co-expression Network Analysis (WGCNA). Gene Ontology (GO) and Kyoto Encyclopedia of Genes and Genomes (KEGG) analyses were conducted to elucidate the functions of the DEGs. PCD-related genes were cross-referenced with the identified DEGs. Diagnostic genes were selected using Least Absolute Shrinkage and Selection Operator (LASSO) and Random Forest (RF) methodologies. Single-cell RNA sequencing was utilized to assess gene expression in blood cells, while CIBERSORT was employed to evaluate immune cell infiltration. A transcription factor (TF)-microRNA (miRNA)-hub gene network was constructed, and potential therapeutic compounds were predicted using the Drug Gene Interaction Database (DGIdb). Mendelian Randomization (MR) methods were applied to analyze genome-wide association study (GWAS) data for S100A9, TXN, and GSTO1.

**Results:**

The analysis revealed 2156 PCD-related genes, 714 DEGs, and 1198 hub genes, with 88 genes enriched in immune and cell death pathways. Five pivotal PCD-related genes (IRAK3, S100A9, TXN, NFATC2, and GSTO1) were identified, leading to the construction of a network comprising six transcription factors and 171 microRNAs. Additionally, seven drugs targeting S100A9, TXN, and NFATC2 were identified. MR analysis suggested that a decrease in GSTO1 levels is associated with an increased risk of sepsis, and that sepsis influences the levels of S100A9, TXN, and GSTO1.

**Conclusions:**

Through bioinformatics approaches, this study successfully identified five genes (IRAK3, S100A9, TXN, NFATC2, and GSTO1) associated with programmed cell death in the context of sepsis. This research identified seven candidate drugs for sepsis treatment and established a methodological framework for predicting biomarkers and drug targets that could be applicable to other diseases.

**Supplementary Information:**

The online version contains supplementary material available at 10.1186/s41065-025-00403-w.

## Introduction

Sepsis is an organ dysfunction caused by a dysregulated host response to infection, which is the main cause of harm to human life and health [1]. A 2017 study showed that there were about 48 million patients with sepsis in the world every year, of which about 11 million died due to sepsis, accounting for 19.7% of the total number of deaths in the world [2]. A large prospective study in China showed that patients with sepsis occupied one fifth of intensive care units (ICUs), and the 90-day mortality rate reached 35.5%. It is estimated that the medical cost of ICU treatment of patients with sepsis in China is about US $4.6 billion per year [3]. Due to its high morbidity, mortality, and significant medical and social burden, sepsis has become a global public health problem. Although the treatment of sepsis has evolved rapidly in the past few years, its morbidity and mortality remain high. Because the exact mechanism of sepsis is still unclear, there is no specific diagnostic or therapeutic index so far, which brings great challenges to the accurate diagnosis and treatment of sepsis.

In recent years, with the development of whole genome sequencing technology, a large number of omics data have been generated. Using these data for bioinformatics analysis to identify disease diagnosis and treatment targets has become a research hotspot. Programmed cell death (PCD) is an important form of cell death, which is closely related to the occurrence and development of many diseases [4]. PCD is a complex regulatory process involving multiple mechanisms in vivo. A total of 15 PCD modes have been found, including pyroptosis, ferroptosis, necroptosis, autophagy, immunogenic cell death, endocytotic cell death, copper death, PARP-1 mediated apoptosis (Parthanatos), lysosome-dependent cell death, endogenous cell apoptosis, exogenous cell apoptosis, cell necrosis, anoikis cell death, apoptosis-like morphology, and necrosis-like morphology [5]. A large number of studies have confirmed that PCD plays a key role in the occurrence and development of sepsis [6, 7]. However, during the occurrence and development of sepsis, different PCD modes may appear simultaneously or successively, and play different roles. At the same time, multiple PCD modes interact to affect the cell outcome. An increasing number of studies have shown that various PCD processes play a key role in the pathophysiology of sepsis and may become new targets for sepsis diagnosis and treatment in the future [8–11].

In this study, we used bioinformatics technology to screen PCD related diagnostic genes and targeted drugs for treatment of sepsis, and verified the related causality using Mendelian randomization analysis, which is conducive to the realization of multi-target comprehensive intervention of sepsis and provides a new option for the prevention and treatment of patients with sepsis.

## Methods

### Data sources

GEO database (http://www.ncbi.nih.gov/geo), the full name is Gene Expression Omnibus, is a database for high-throughput gene expression datacreated by the National Center for Biotechnology Information (NCBD) in 2000 and maintained today. We downloaded the GSE28750, GSE57065, GSE95233 and GSE69063 human sepsis general transcriptional profiles from the GEO database using the GEO query package of R software (version 4.2.1). GSE69063 was used as the validation set. The GSE28750 dataset included 10 sepsis samples and 20 healthy control samples, and the platform was GPL570 [HG-U133_Plus_2] Affymetrix Human Genome U133 Plus 2.0 Array. The GSE57065 dataset included 82 sepsis samples and 25 healthy control samples. The platform was GPL570 [HG-U133_Plus_2] Affymetrix Human Genome U133 Plus 2.0 Array. The GSE95233 dataset included 102 sepsis samples and 22 healthy control samples on the GPL570 [HG-U133_Plus_2] Affymetrix Human Genome U133 Plus 2.0 Array) platform. The platform for the GSE69063 dataset was GPL19983[HuGene21st_Hs_ENTREZG_19.0.0] [HuGene-2_1-st] Affymetrix Human Gene 2.1ST Array. The data set contained 11 healthy control samples, 10 mild sepsis samples and 10 severe sepsis samples. All the three groups of samples were blood samples collected from the first arrival at the emergency department. In addition, we obtained the GSE167363 data set (GPL24676 platform), a single-cell RNA sequencing of human peripheral-blood mononuclear cells from healthy controls and patients with sepsis, from the GEO database.

Genes related to 15 PCD modes were manually collected from MSigDB (http://software.broadinstitute.org/gsea/msigdb/index.jsp), KEGG, FerrDb V2 database (http://www.zhounan.org/ferrdb/current/), Genecards website (https://www.genecards.org/).

A large genome-wide association study (GWAS) with 5,368 participants revealed 4,035 independent associations between genetic variants and 2,091 serum proteins, providing new insights into the interplay between genetics, serum protein levels, and complex diseases [12]. GWAS data for S100A9, TXN, and GSTO1 were available from the publicly available data. The data about S100A9 were obtained from the IEU OpenGWAS database (https://gwas.mrcieu.ac.uk/) prot-a-2622. GWAS data on sepsis were obtained from the Finnish database (https://www.finngen.fi/).

### Identification of differentially expressed genes (DEGs)

Raw GSE57065 data for sepsis were downloaded as MINiML files from the GEO database. Probes were converted to gene symbols based on the platform labeling information of the normalized data. Probes containing more than one gene were eliminated, and the average of genes corresponding to multiple probes was calculated. The limma package in R language (version 4.2.1) was used to obtain DEGs between the two groups. Adjusted *p*-values were calculated to correct for false positives in the GEO dataset. Adjusted *P* < 0.05 and log (fold change) > 1 or log (fold change) < − 1 were defined as the threshold for differentially expressed genes in sepsis. The Complex Heatmap and ggplot2 programs in R language were used to draw heat maps and volcano maps.

### Weighted gene co-expression network analysis (WGCNA)

Raw GSE28750 data for sepsis were downloaded as MINiML files from the GEO database. Based on the platform labeling information of the normalized data, probes were converted to gene symbols and genes containing more than one probe were removed. The median absolute deviation (MAD) was calculated separately for each gene, and the top 50% of genes with the smallest MAD were excluded. The outliers were removed using the “Good Samples Genes” method of WGCNA in R language, and WGCNA was further used to construct a scale-free co-expression network. The principle is to divide the genes with high topological overlap in each sample into a gene module, and then correlate with the occurrence of sepsis on a gene module basis, so as to screen the hub genes in the optimal module. First, Pearson correlation analysis and average linkage analysis were performed for all paired genes. Then, using the power function A_mn =| C_mn| ^ β to construct weighted adjacency matrix, including C_mn represent the Pearson correlation between the gene of m and n, A_mn represents the adjacency relation between gene m and n, beta is a soft threshold parameter, to emphasize the correlation between gene. After selecting the appropriate value of β, the adjacency relationship is transformed into the topological overlap matrix (TOM), which can measure the network connectivity of a gene, which is defined as the sum of adjacency relationships between the gene and all other genes in the network, and the corresponding dissimilarity (1-TOM) is calculated. To partition genes with similar expression profiles into gene modules, average linkage hierarchical clustering was performed according to TOM-based dissimilarity measures, and the minimum value of the gene dendrogram was set to 30. The modules most associated with sepsis were identified as sepsis-related modules to screen hub genes. The screening criteria were: The Pearson correlation coefficient between the expression level of each gene and clinical features was Gene Significance (GS) > 0.2, and the Pearson correlation coefficient between the characteristic genes of each Module and clinical features was Module Significance (MS) > 0.2. The Pearson correlation coefficient between the genes in the module and the characteristic genes of the module was Module Memberships (MM) > 0.8.

### Functional and pathway enrichment analyses

The Venn diagram drawing tool (http://bioinformatics.psb.ugent.be/webtools/Venn/) was used to generate Venn diagrams of DEGs, WGCNA hub genes, and PCD related genes to obtain crossover genes. To further confirm the potential functions of genes, we performed functional and pathway enrichment analyses on the cross-gene data obtained above. In this study, the ClusterProfiler package (version 3.18.0) in R language was used to perform Gene Ontology (GO) function and Kyoto Encyclopedia of Genes and Genomes (KEGG) pathway enrichment analysis of potential targets. GO enrichment analysis is a tool for functional gene annotation, which has been widely used in recent years and includes three parts: molecular function (MF), biological pathway (BP) and cell component (CC). KEGG enrichment analysis is a practical resource for studying gene function and related high-level genomic functional information. Heat maps were drawn using the R language package pheatmap. Gene set enrichment analysis (GSEA) was used to further analyze the pathway regulation. We use the KEGG rest API (https://www.kegg.jp/kegg/rest/keggapi.html) for the latest gene annotations. In this analysis, the minimum gene set was 5 and the maximum gene set was 5000. *P* values < 0.05, FDR < 0.25 and|NES| > 1, were considered significant.

### Identification and validation of diagnostic marker genes

Least Absolute Shrinkage and Selection Operator (LASSO) regression can compress the variable coefficients to prevent overfitting and address severe collinearity. Random Forest (RF) algorithm was used to select diagnostic marker genes, and the weight information of each gene was provided. The features are ranked according to their weight, with higher weight features being more important. Hiplot Pro is an integrated web service for biomedical data analysis and visualization. After normalization of sepsis GSE95233 dataset, LASSO analysis and random forest analysis were performed using the tools in Hiplot Pro (https://hiplot.com.cn/) to obtain diagnostic marker genes. The Venn diagram drawing tool was then used to generate common genes for both analyses.

To validate the diagnostic marker genes, we analyzed the differences in gene expression in the sepsis dataset GSE69063. Kolmogorov-Smirnov test was used to compare data between multiple groups, and *P* value < 0.05 was considered statistically significant. Meanwhile, based on univariate logistic regression, the obtained common genes were included to draw the Nomogram. The pROC R 1.17.0.1 software package was used to draw receiver operating characteristic (ROC) curve and calculate the area under ROC curve (AUC) to detect the diagnostic value of marker genes for sepsis.

### Single-cell RNA-seq analysis

Principal component analysis (PCA) and t-Distributed Stochastic Neighbor Embedding (t-SNE) were performed on the single-cell dataset GSE167363 using the “Seurat” R package. Those cells with more than 4000 features, more than 25% mitochondrial genes, or fewer than 200 features were excluded. After data dimensionality reduction, to normalize each cell, we scaled the UMI counts using scale factor = 10,000. After log transformation of the data, variant regression was corrected using the ScaleData function (v3.0.2) in Seurat. The corrected normalized data were applied to the standard analysis. PCA was performed, and we identified significant principal components by the elbow method. We performed cell clustering using the FindClusters function (resolution = 0.5) implemented in the Seurat R package. We chose “Human Primary Cell Atlas Data” as a reference data set. Violin plots are used to show the expression of diagnosis-related genes in different blood cells.

### Analysis of immune cell infiltration

In order to compare sepsis and the change of immune cell proportion in the healthy controls, using CIBERSOTR algorithm (http://cibersort.stanford.edu/), analyze the GSE28750 sequencing data, determined the abundance of 22 kinds of immune cell subtype. These subtypes represent the cellular composition of the septic immune microenvironment, and differences in the proportion of immune cells were calculated using the Wilcoxon rank-sum test, with *P* < 0.01 considered significant. And the Spearman correlation analysis was carried out on the genes and immune cells, setting the threshold value is: *P* < 0.05 and|r| > 0.6. The pheatmap package was applied to visualize the results.

### Construction of transcription factor (TF)-microRNA-hub gene network

MicroRNAs (miRNAs) and TFs associated with cross-over genes were identified using the miRNet2.0 online database (https://www.mirnet.ca/) and plotted using Cytoscape software (version 3.8.2).

### Screening of potential therapeutic drugs for sepsis

Biomarker genes were identified using the DGIdb database. As a database of common drug-gene interactions, DGIdb (https://www.dgidb.org/) brings together drug-gene information from various resources.

### Search for instrumental variables

Single Nucleotide Polymorphisms (SNPs) associated with S100A9, TXN and GSTO1 were selected as instrumental variables in forward Mendelian randomization (MR) analysis.

The instrumental variables should meet the following criteria: (1) *P* < 5 × 10^− 6^ was used as the screening criterion to obtain data on SNPs associated with exposure factors of S100A9, TXN and GSTO1. (2) Linkage disequilibrium (LD) among instrumental variables was eliminated (the region range of the screened instrumental variables was 10,000 kb, and SNPs with LD parameter r^2^ > 0.001 should be excluded to ensure the independence of the screened instrumental variables). (3) SNPs associated with S100A9, TXN and GSTO1 were identified from the sepsis as outcome database (The PhenoScanner database was used to exclude SNPs that did not meet the criteria, confounding factors (such as infection, pancreatitis, severe trauma, etc.) related SNPs, and SNPs with missing information). (4) Weak instrumental variables were removed according to F-test, and F > 10 was the screening threshold.

Sepsis was set as the exposure, while S100A9, TXN, and GSTO1 were set as outcomes in the reverse MR Analysis. The same screening criteria as for forward MR were set to obtain SNPs that were associated with sepsis on a genome-wide scale and without LD.

### Validation of instrumental variables

Both the forward and reverse MR study must satisfy the following three assumptions: (1) the instrumental variables are associated with S100A9, TXN and GSTO1; (2) the instrumental variables only affect sepsis through S100A9, TXN and GSTO1, but do not directly affect sepsis; (3) the instrumental variables were independent of the confounders associated with S100A9, TXN and GSTO1 and sepsis risk.

Pleiotropy test: MR-Egger regression can test for pleiotropy in Mendelian randomization analyses, and the strength of the assessment instrument does not rely on the null causality hypothesis under the direct effect hypothesis. The principle is to use the inverse variance of the outcome for weighted calculation, and the intercept term is included in the regression, without requiring the line to pass through the origin [13]. Therefore, applying MR-Egger regression is able to provide unbiased estimates even if all selected SNPs are null.

Elimination of confounding factors: If SNPs influence sepsis through confounding factors, they will confound the outcome of Mendelian randomization. Therefore, in order to minimize the influence of confounding factors on sepsis, these SNPs will be further examined in the PhenoScanner database, and SNPs that affect the outcome of sepsis by interacting with known confounding factors (such as infection, pancreatitis, severe trauma, etc.) will be excluded.

### MR analysis

Two Sample MR Software in R was used as the main statistical analysis software, and the test level was α = 0.05. According to the β and standard error (SE) values between instrumental variables and exposure factors, as well as between instrumental variables and outcome risk, multiple methods for estimating causal effects were used after integration to study the causal relationship between S100A9, TXN, GSTO1 and sepsis risk. This study mainly uses the proposed Inverse-variance Weighting (IVW) method for estimation. In order to improve the accuracy of the study, this paper further uses MR-Egger regression, Weighted Median (WM) method, Weighted Mode and Simple-mode to supplement the causal association between exposure factors and outcomes.

### Sensitivity analysis

IVW and MR-Egger regression were used to assess the heterogeneity of the analysis results, and Q test was used to quantify the heterogeneity. A threshold of *P* > 0.05 indicated that there was no heterogeneity in the selected instrumental variables. Leave-one-out sensitivity analysis was used to determine the potential impact of SNPs on causal effects, and the results of sensitivity analysis were presented in the form of forest plots.

## Results

### Screening of DEGs and weighted gene co-expression network analysis (WGCNA)

**Fig. 1:**
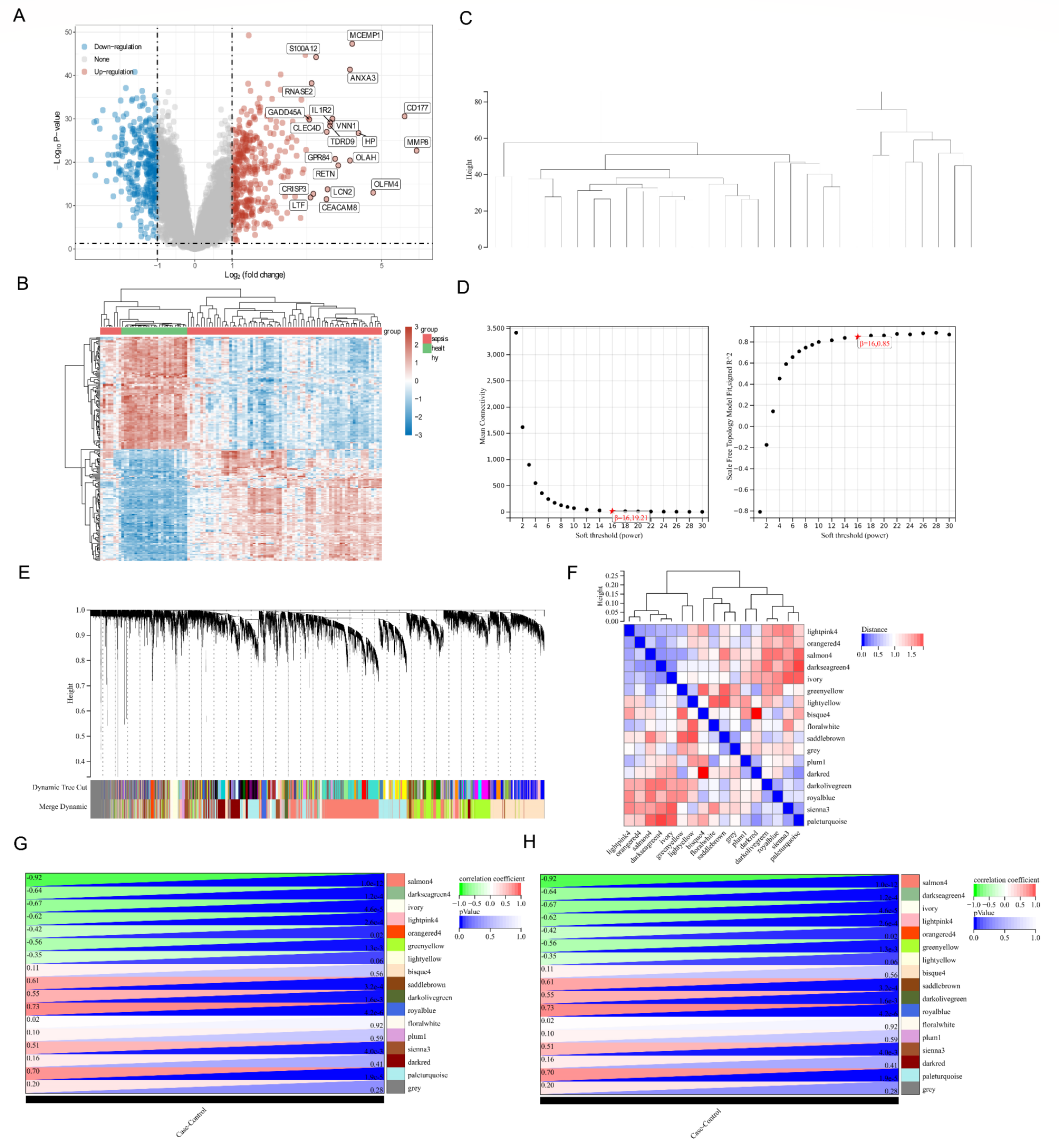
DEGs and WGCNA analysis. **A**: Volcano plot of the DEGs in the GSE57065 dataset; **B**: Heatmap of the DEGs in the GSE57065 dataset; **C**: Clustering trees for each dataset; **D**: Analyze of network topology for various soft-thresholding powers; **E**: Clustering dendrogram of the genes; **F**: The adjacencies of modules in the network; **G**: Heatmap of the correlation between module and sepsis; **H**: Scatter plots of GS for sepsis versus MM in the salmon4 module

In this study, 2156 genes related to 15 PCD modes were manually collected from databases and previous review articles. The number of genes and partial gene names are listed in Table 1. Subsequently, through processing the GSE57065 dataset we found that 714 genes were differentially expressed in sepsis compared with normal controls, 389 genes were up-regulated and 325 genes were down-regulated. Differentially expressed genes between sepsis and control were displayed using volcano plots (Fig. 1A) and heat maps (Fig. 1B).

**Table 1:** Information about genes related to different kinds of programmed cell death

PCD	GENE COUNTS
Pyroptosis	388 (GSDMD, GSDME, NLRP3, CASP1, CASP4, GSDMB, etc.)
Parthanatos	23 (AIFM1, PARP1, ADPRS, RNF146, NAMPT, GPX4, MAPK8, etc.)
Necrosis	500 (TNF, COL2A1, TNFRSF1B, IL6, IL1B, FAS, etc.)
Necrosis like morphology	73 (TP53, AKT1, VIM, MYC, BCL2, KRT5, RAC1, FAS, ABL1, etc.)
Necroptosis	500 (TP53, MEFV, AIM2, TNFAIP3, UCHL1, STING1, TNIP1, etc.)
Lysosome dependent cell death	194 (EGFR, ATM, AKT1, TNF, PDCD1, MTOR, IL6, VHL, etc.)
Intrinsic apoptosis	500 (CD27, JAK3, LCK, IER3IP1, JAK1, RAC1, etc.)
Immunogenic cell death	500 (TP53, ADA, FAS, EGFR, BCL2, PTEN, CASP8, ATM, etc.)
Ferroptosis	283 (GPX2, GPT2, GPAT4, GLUT13, GDF15, GCH1, etc.)
Extrinsic apoptosis	500 (BCL2, CASP3, FAS, BAX, XIAP, CFLAR, etc.)
Entotic cell death	23 (TP53, MTOR, TNFSF10, GZMB, EZR, MRTFA, DIAPH1, etc.)
Cuproptosis	17 (ATP7A, ATP7B, CDKN2A, DBT, DLAT, DLD, DLST, FDX1, etc.)
Autophagy	222 (BAX, BCL2, IL24, TP63, TP73, TSC1, TSC2, etc.)
Apoptosis like morphology	146 (JAK2, AR, TUBB, NDE1, GLI1, CYCS, etc.)
Anoikis	27 (PDK4, TSC2, PTRH2, STK11, NOTCH1, TLE1, CAV1, etc.)

A total of 20,535 genes were obtained from 30 samples of the GSE28750 dataset. These genes were used to construct a co-expression network. The results of cluster analysis of the samples are shown in Fig. 1C. Cluster trees were built for each dataset and no outliers were found. The scale-free fit index and mean connectivity were calculated, and a power of β = 16 (scale-free R^2^ = 0.85) was chosen (Fig. 1D). The minimum number of genes per module was set to 30 according to the criteria of the dynamic tree cutting algorithm. Finally, 17 transcription modules indicated by distinct colors were identified (Fig. 1E). The correlation heatmap of the modules in the network is shown in Fig. 1F. To correlate the modules with the sample information, we correlated the clinical feature data by analyzing the data according to the module-clinical feature correlation heatmap (Fig. 1G). The light orange 4(salmon4) module, which was identified as a hub module associated with clinical traits, was used to explore the correlation between module membership (MM) and gene significance (GS) to identify hub genes for sepsis (Fig. 1H). As a result, 1198 hub genes were identified from the salmon4 modules described above and used for subsequent analyses.

### Analysis of cross genes function and pathway enrichment

**Fig. 2:**
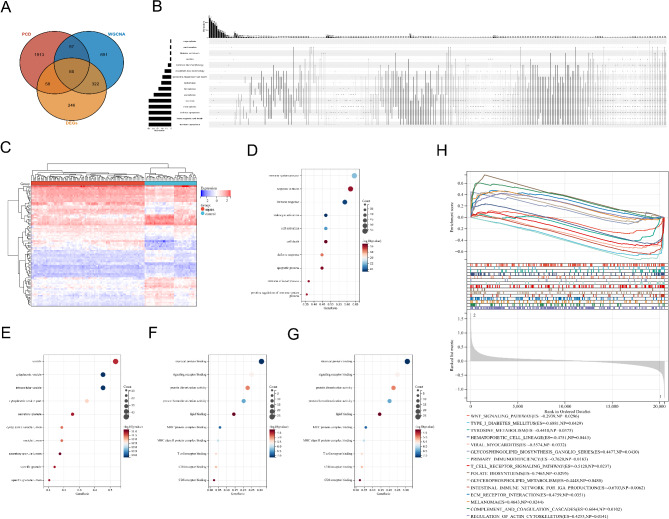
Function and pathway enrichment analysis of cross-genes. **A**: Venn diagram of PCD-related genes, WGCNA-related genes and DEGs; **B**: Upset of 15 PCD-related genes; **C**: Heatmap of the 88 DEGs in the GSE95233 dataset; **D**: Biological process of DEGs; **E**: Cellular component of DEGs; **F**: Molecular functions of DEGs; **G**: KEGG enrichment analysis of DEGs; **H**: GSEA for GSE28750

The 2156 genes related to 15 PCD modes and 1198 hub genes obtained by WGCNA were crossed with 714 DEGs from GSE57065 dataset to draw a Venn diagram. Eighty-eight overlapping DEGs and were used for subsequent further analysis, as shown in Fig. 2A. The names of these 88 overlapping genes are given in Supplemental materials. At the same time, the genes among the 15 PCD modes also overlapped. In order to better display this cross-connection, we used online tools to draw Upset maps of the 15 PCD modes, as shown in Fig. 2B. These 88 cross DEGs in sepsis and control groups were shown using a heat map (Fig. 2C).

Based on the 88 DEGs associated with PCD obtained in the above studies, we performed KEGG and GO enrichment analyses. The analysis results of the top 10 GO terms showed that the genes common in sepsis were mainly enriched in biological processes such as immune response, leukocyte activation, cell death, immune system defense and regulatory response, apoptosis, and immune effector process (Fig. 2D). The cellular component enriched in the main localization in cells included cytoplasmic vesicles, intracellular vesicles, and secretory granules (Fig. 2E). Molecular functions mainly include signal receptor binding, protein homodimerization activity, lipid binding, Major Histocompatibility Complex (MHC) protein complex binding, T cell receptor binding, CD4 receptor binding, CD8 receptor binding, and so on (Fig. 2F). KEGG analysis showed that sepsis genes were mainly involved in T cell receptor signaling pathway, Th1 and Th2 cell differentiation, hematopoietic cell lineage, Th17 cell differentiation, cell apoptosis, NF-κB signaling pathway, natural killer cell-mediated cytotoxicity, primary immunodeficiency, and so on (Fig. 2G). At the same time, a large number of cross-relationships among genes, biological functions and pathways were found, indicating that the role of the above genes in sepsis is comprehensively regulated through a complex biological function and pathway network.

Gene set enrichment analysis (GSEA) showed that the identified KEGG pathways in sepsis compared to controls were Wnt signaling pathway, tyrosine metabolism, T cell receptor signaling pathway, viral myocarditis, folate synthesis, and regulation of actin-cytoskeleton (Fig. 2H).

### Identification and validation of diagnostic marker genes

**Fig. 3:**
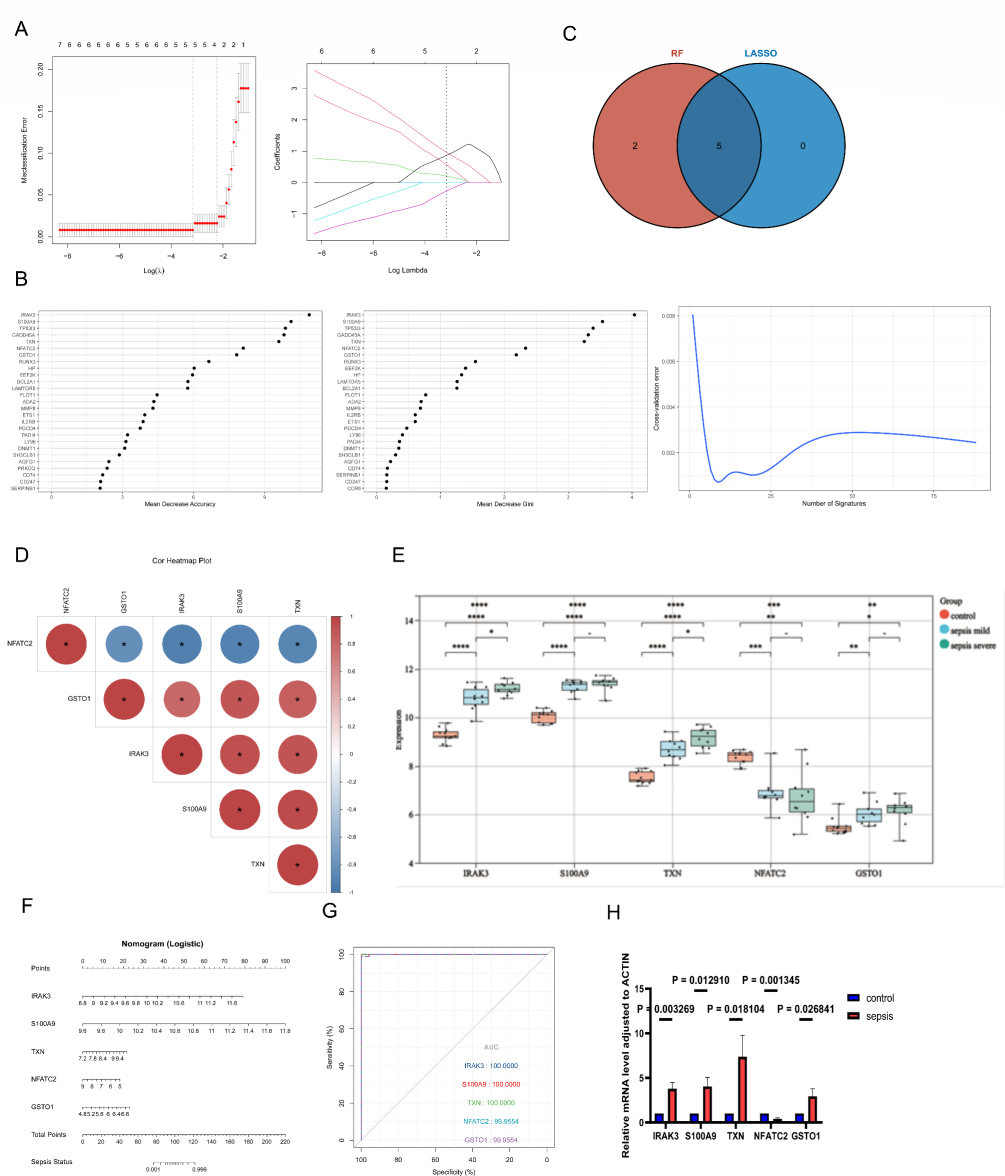
Identification and validation of diagnostic marker genes. **A**: LASSO analysis of sepsis; **B**: Random Forest analysis of sepsis; **C**: Venn diagram of LASSO analysis and Random Forest analysis; **D**: Correlation heatmap of 5 genes; **E**: The expression of 5 genes in GSE69063; **F**: Nomogram to predicting the risk of common genes in sepsis patients; **G**: Quantification of ROC curves values for diagnostic model constructed by 5 key genes; **H**: The expression of 5 genes in healthy and sepsis patients (* *P* < 0.05; ** *P* < 0.01; *** *P* < 0.001)

LASSO analysis and the random forest method were used to screen 88 DEGs closely related to sepsis marker genes. LASSO analysis obtained 5 marker genes (Fig. 3A), and random forest method obtained 7 marker genes. A goodness-of-fit plot generated by the random forest algorithm showed its stability and high accuracy (Fig. 3B). The Venn diagram drawing tool was further used to generate five key genes for the two analyses, namely IRAK3, S100A9, TXN, NFATC2 and GSTO1(Fig. 3C). The correlation heat map showed significant associations between these five genes (Fig. 3D).

In order to verify the validity of the diagnostic marker genes, a KS test was performed on the GSE69063 dataset for these five genes, and the results showed that IRAK3, S100A9, TXN and GSTO1 were up-regulated in the sepsis group, while NFATC2 was down-regulated. The expression of IRAK3 and TXN in the control group, mild sepsis group and severe sepsis group increased in a gradient manner (Fig. 3E). Based on univariate logistic regression analysis, the above five genes were included in the Nomogram, as shown in Fig. 3F. The diagnostic model constructed by the five key genes also showed excellent performance, and the area under the ROC curve AUC was close to 1, *P* = 0.043(Fig. 3G).

To determine the expression levels of diagnostic genes in the Chinese population in the sepsis group and the normal control group, IRAK3, S100A9, TXN, NFATC2 and GSTO1 were validated using RT-PCR with peripheral blood samples from clinical patients. The results showed that the expression of IRAK3, S100A9, TXN and GSTO1 in the sepsis group was increased, while the expression of NFATC2 in the sepsis group was decreased (*P* < 0.05, Fig. 3H).

### Single-cell RNA-seq analysis

**Fig. 4:**
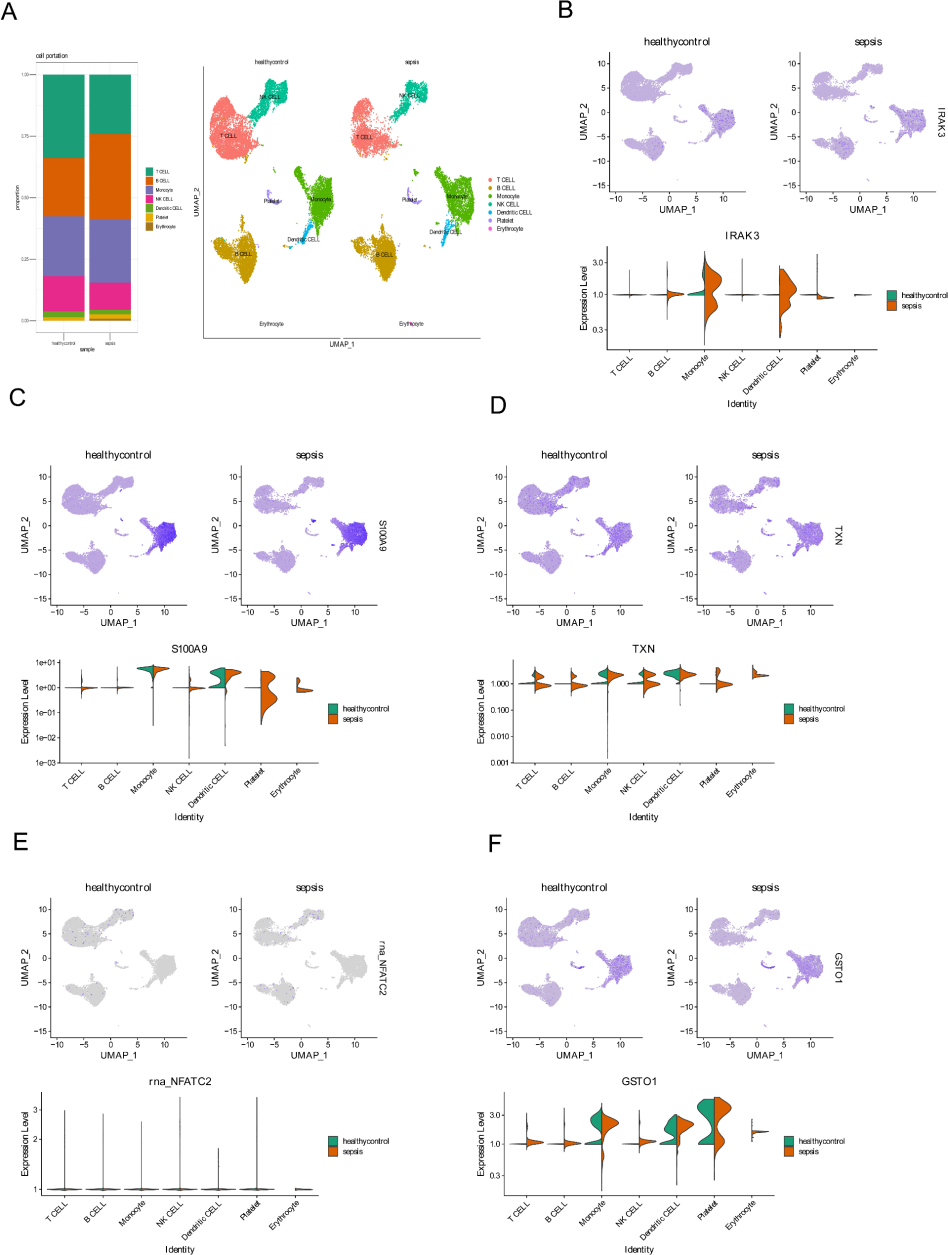
Single-cell RNA-seq analysis. **A**: Single-cell subpopulation identification and expression levels of diagnostic genes in Sepsis and normal groups in GSE167363 database; expression of IRAK3 (**B**), S100A9(**C**), TXN(**D**), NFAC2(**E**), and GSTO1(**F**) in blood cell types based on the GSE167363 database

To determine the single-cell subsets and expression levels of diagnostic genes in the sepsis and normal groups, we downloaded data from the GSE167363 database. The analysis revealed decreased T cells and NK cells and increased B cell subsets in the sepsis group (Fig. 4A). Cells were clustered using the t-SNE algorithm, yielding 21 clusters (Fig. 4A).

Figure 4B shows the distribution and expression of IRAK3 in different cell types; IRAK3 was highly expressed in monocytes and dendritic cells in the sepsis group. S100A9 was expressed in a variety of cell types, and was higher in NK cells from the sepsis group than in the control group (Fig. 4C). TXN was also expressed in a variety of cell types, with higher expression in B cells in the sepsis group than in the control group (Fig. 4D). NFATC2 expression was also lower in multiple cell types (Fig. 4E). GSTO1 was also expressed in a variety of cell types, and the expression of GSTO1 in T cells, B cells and NK cells in the sepsis group was higher than that in the control group (Fig. 4F).

### Analysis of immune cell infiltration

**Fig. 5:**
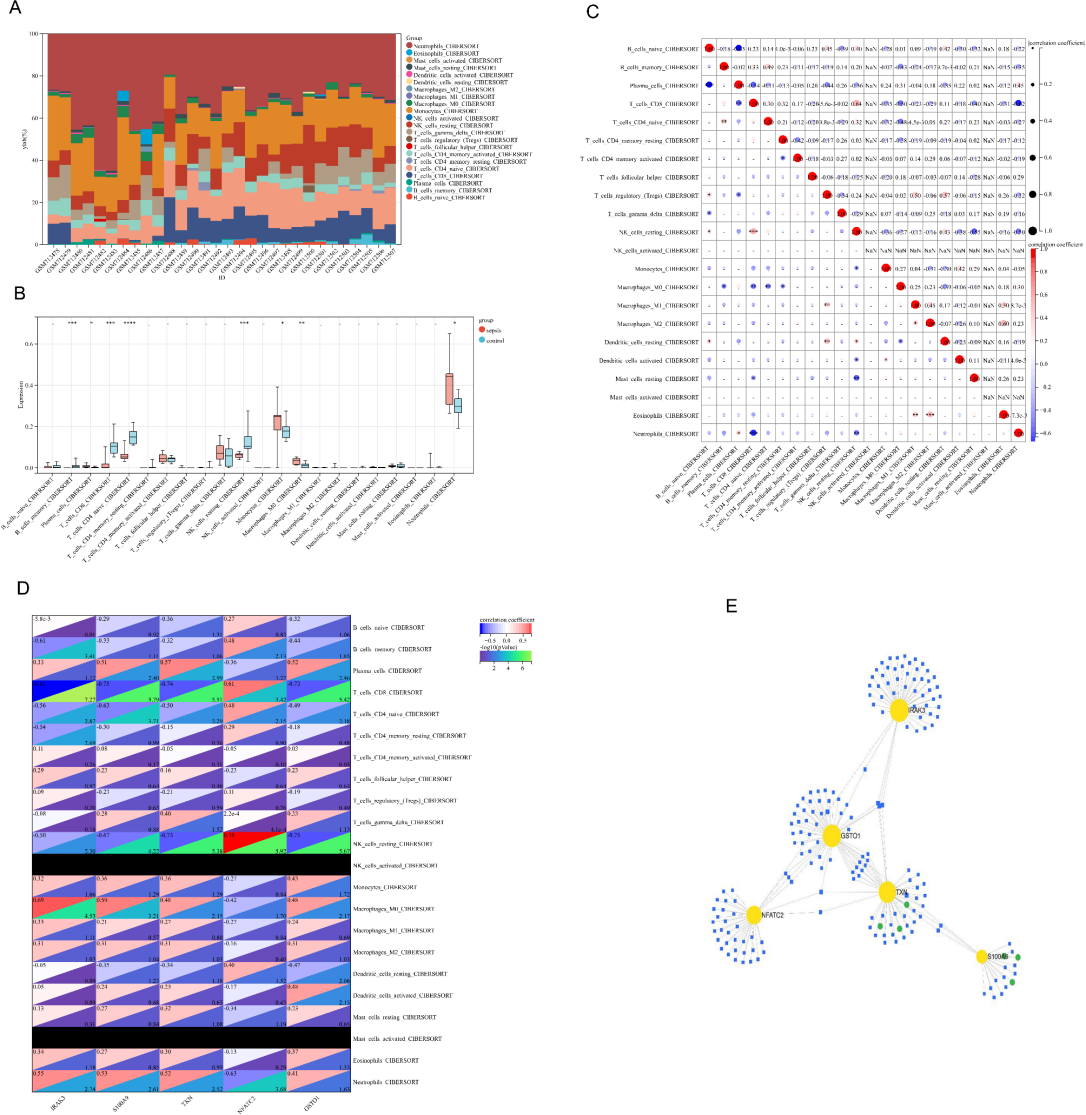
Immune cell infiltration analysis and transcription factor-microRNA-hub gene network. **A**: Stacked histogram displaying the immune cell proportions between sepsis and control groups; **B**: Violin plot showing the comparison of 22 kinds of immune cells between sepsis and control groups; **C**: The heatmap revealing the correlation of 22 kinds of immune cells infiltration; **D**: The correlation map representing the association of the differentially infiltrated immune cells with 5 hub genes; **E**: Construction of the TF-miRNA-hub gene network in sepsis based on miRnet (* *P* < 0.05; ** *P* < 0.01; *** *P* < 0.001)

We found that functional and pathway analyses of sepsis-related genes were closely related to inflammatory and immune processes. The CIBERSOTR algorithm was used to derive the characteristics of immune cells, and to explore the correlation between immunomodulatory and diagnostic biomarkers and immune cell infiltration in sepsis. Figure 5A shows the proportion of 22 immune cells in each sample, and there were significant differences between the sepsis group and the control group in eight immune cell subsets. Compared with the control group, the sepsis group showed a higher proportion of monocytes, macrophages M0 and neutrophils. In contrast, the proportions of memory B cells, CD8 + T cells, CD4 + T cells, and resting NK cells were reduced (Fig. 5B). In addition, correlation analysis of 22 immune cells showed that CD8 + T cells were positively correlated with resting NK cells (*r* = 0.64, *P* < 0.05), and CD8 + T cells were negatively correlated with neutrophils (*r* = -0.62, *P* < 0.05) (Fig. 5C). We also explored the relationship between the expression of five key genes and the proportion of different immune cell types, and CD8 + T cells and resting NK cells were significantly associated with five key genes in sepsis (Fig. 5D).

### Construction of TF-miRNA-Hub gene network and screening of potential therapeutic drugs for sepsis

We further investigated the regulatory mechanisms of these five genes in sepsis. The target miRNAs and transcription factors (TFs) of 5 genes were identified (Table S1), and the TF-miRNA-hub gene network was constructed based on miRnet. Finally, we constructed a network containing five genes, six TFs, and 171 miRNAs. Through the complex regulatory network, we can observe the regulation of multiple miRNAs corresponding to a gene, and different genes can also share the same miRNA (Fig. 5E).

Using DGIdb, we screened for potential therapeutic compounds related to five marker genes in sepsis. Among them, compounds corresponding to IRAK3 and GSTO1 genes were not found. Ultimately, we identified seven compounds as potential treatments for sepsis (Table 2).

**Table 2:** The potential compounds of three genes were identified using DGIdb

Gene	Drug	Query Score	Interaction Score
S100A9	Tasquinimod	8.61	61.83
	Paquinimod	4.3	30.91
TXN	PX-12	8.61	30.91
	Gambogenic acid	4.3	15.46
	Biotiny lated gambogic acid	4.3	15.46
	Gambogic acid	0.96	3.43
NFATC2	Asparaginase	0.57	8.24

### Mendelian randomization (MR) analysis

In the pooled GWAS data of S100A9, TXN and GSTO1, significant and independent SNPs were identified (*P* < 5 × 10^− 6^, r^2^ = 0.001, F-test > 10). Thirty-eight SNPs were preliminarily screened as instrumental variables for S100A9 (Table S2), 23 SNPs as instrumental variables for TXN (Table S3) and 18 SNPs as instrumental variables for GSTO1 (Table S4).

**Fig. 6:**
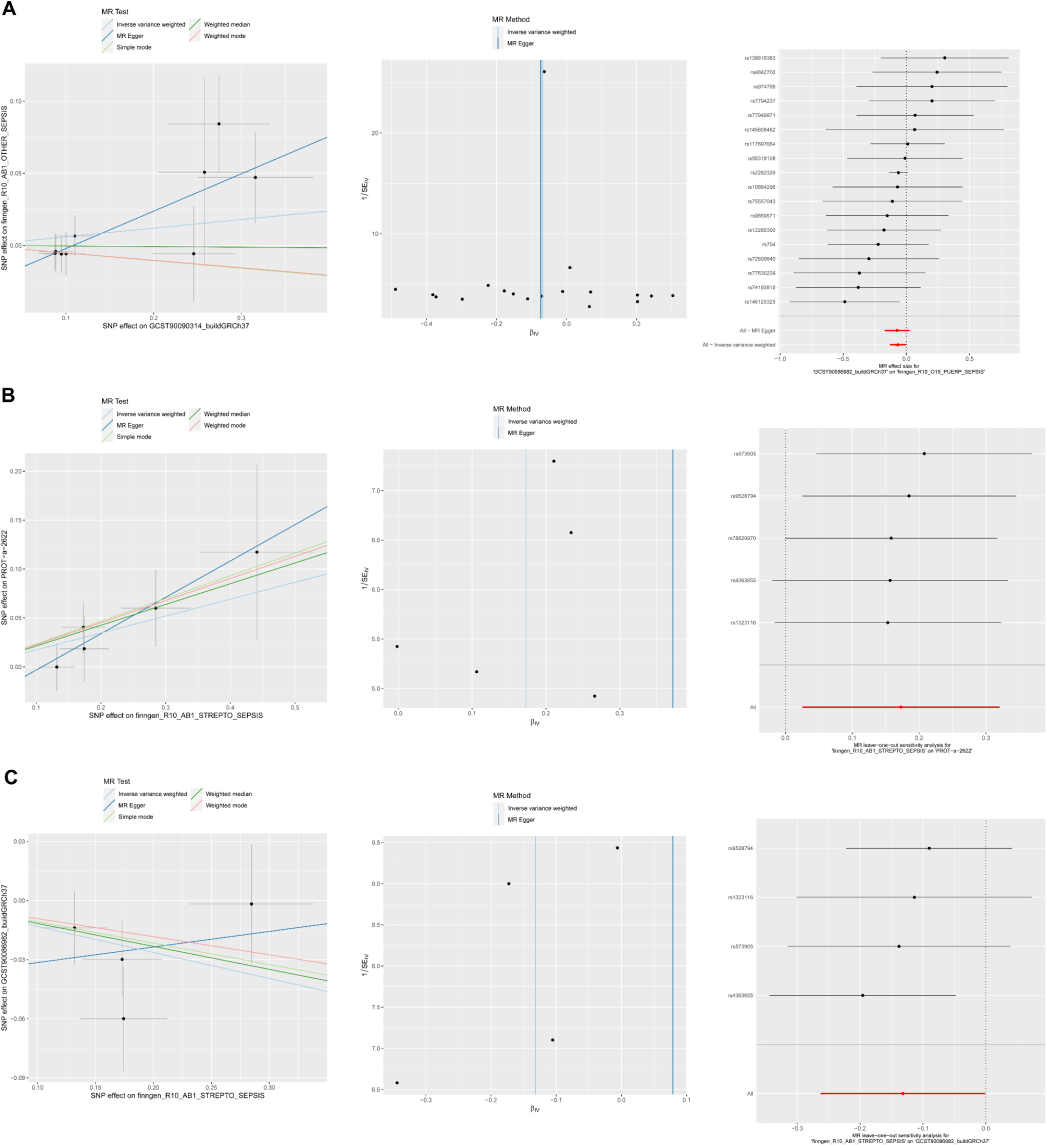
Mendelian randomization analysis. **A**: The scatter, funnel and leave-one-out plots of GSTO1; The scatter, funnel and leave-one-out plots of sepsis on S100A9 (**B**) and GSTO1 (**C**)

Using the “Two Sample MR” software package for analysis, IVW results in Table 3 showed that there was no statistically significant difference in the relationship between S100A9 and TXN levels and sepsis. As shown in Fig. 6A, lower GSTO1 levels were associated with a higher risk of sepsis (OR = 0.93, 95%CI: 0.87–0.99, *P* = 0.034). Among them, the difference between MR-Egger’s intercept term (Fig. 6A) and zero was not statistically significant (*P* > 0.05), indicating that there was no horizontal pleiotropy on its surface. The heterogeneity between SNPS was not statistically significant (*P* > 0.05). The funnel plot showed that the instrumental variables representing causal association effects were roughly symmetrically distributed. The sensitivity analysis of “leave-one-out” method showed that after excluding a single SNP, the IVW analysis results of the remaining SNPS did not change significantly. Therefore, SNPs that influence the causal association estimates to be large do not exist.

**Table 3:** The analysis of the relationship between S100A9, TXN, GSTO1 and sepsis

Exposure	Outcome	Method	Nsnp	β	SE	OR(95%)	*P* value
S100A9	sepsis	IVW	14	0.109048	0.079937	1.11(0.95 ∼ 1.3)	0.17
TXN	sepsis	IVW	13	-0.043613	0.051022	0.95(0.87 ∼ 1.06)	0.81
GSTO1	sepsis	IVW	18	-0.068064	0.032063	0.93(0.87 ∼ 0.99)	0.034

Note: Nsnp: numbers of single-nucleotide polymorphism, and SE: standard error of Beta

The IVW results of Table 4 showed that the occurrence of sepsis had an impact on the levels of S100A9, TXN and GSTO1, with statistically significant differences (*P* < 0.05). Figure 6B and C show the scatter, funnel and leave-one-out plots of sepsis on S100A9 and GSTO1, respectively. Among them, the difference between the intercept term of MR-Egger and zero was not statistically significant (*P* > 0.05), indicating that there was no horizontal pleiotropy on its surface. The heterogeneity between SNPs was not statistically significant (*P* > 0.05). Funnel plots showed that the instrumental variables representing causal association effects were roughly symmetrically distributed. The sensitivity analysis of “leave-one-out” method showed that after excluding a single SNP, the IVW analysis results of the remaining SNPS did not change. Therefore, SNPs that influence the causal association estimates to be large do not exist. Unfortunately, further analytical tests could not be performed due to the small number of instrumental variables obtained for TXN protein.

**Table 4:** The analysis of the relationship between sepsis and S100A9, TXN, GSTO1

Exposure	Outcome	Method	Nsnp	SNP	β	SE	*P* value
sepsis	S100A9	IVW	5	rs1323116	0.172952	0.762969	4.19E-07
				rs4363655	0.284632	0.063208	1.08E-07
				rs573905	-0.13181	0.558107	2.81E-06
				rs78620970	0.441019	0.019533	4.34E-07
				rs9528794	0.174185	0.813407	3.91E-06
sepsis	TXN	IVW	1	rs1980496	0.083082	0.295848	1.06E-09
sepsis	GSTO1	IVW	4	rs1323116	0.172952	0.762969	4.19E-07
				rs4363655	0.284632	0.063208	1.08E-07
				rs573905	-0.131809	0.558107	2.81E-06
				rs9528794	0.174185	0.813407	3.91E-06

Note: SNP: single nucleotide polymorphism, Nsnp: numbers of SNP, and SE: standard error of Beta

## Discussion

Sepsis is caused by the body’s uncontrolled immune response to infection, resulting in shock and multiple vital organ dysfunction syndrome, with high mortality and lack of specific diagnostic criteria. At present, the research on accurate and early diagnosis and effective treatment of sepsis is a global concern [14]. Patients in the initial high inflammatory phase of sepsis survive as aggressive source control, early appropriate antibiotic therapy, titrant and compression therapy, and better organ support measures, especially ventilator management, have improved clinical outcomes over the past decade. As sepsis progresses to the later stage of immunosuppression, 30% of these patients still die from secondary infections. Therefore, early identification and prediction of patients with acute inflammatory infections who are at risk of sepsis, including sepsis-related adverse outcomes, are essential. In recent years, with the development of whole genome sequencing technology, a vast amount of omics data has been generated, and the use of these data for bioinformatics analysis to find disease diagnosis and therapeutic targets has become a research hotspot [15]. Programmed cell death is essential for organ development, tissue homeostasis, and tissue injury and tumorigenesis. Abnormalities in the process of programmed cell death are associated with a variety of human diseases, including sepsis [16].

In this study, we collected genes associated with PCD in 15 patterns, which is more comprehensive than previous studies. The genes encoding 5 diagnostic biomarkers (IRAK3, S100A9, TXN, NFATC2 and GSTO1) were identified. Interleukin-1 receptor associated kinase 3 (IRAK3) is a key regulator of inflammation and has been linked to endotoxin tolerance and sepsis. Although IRAK3 is known to be a negative regulator of inflammation, However, several studies have reported its opposite function [17, 18], and the role of IRAK3 on inflammation remains unclear. In a systematic review and meta-analysis, it was found that IRAK3 expression and its effect on inflammatory markers (TNF-α and IL-6) varied at different stages of sepsis, affected by changes in experimental procedures [19]. IRAK3 can promote cell apoptosis [20], and IRAK3 has been identified as a pyrogenic gene in hypertrophic cardiomyopathy [21]. Our analysis showed that the expression of IRAK3 increased in the control group, mild sepsis group and severe sepsis group in a gradient pattern, which was consistent with the results of previous literature. S100 calcium binding protein A9 (S100A9) is a Ca^2+^-binding protein of the S100 family, which usually exists in the form of heterodimers with S100A8 and is mainly derived from immune cells. S100A9 can promote apoptosis [22] and inhibit pyroptosis [23]. It has been found that S100A8/A9hi neutrophils induce endothelial mitochondrial dysfunction and PANoptosis (a new type of programmed cell death that combines the features of apoptosis, necroptosis, and pyroptosis) in sepsis [24]. Thioredoxin (TXN) is a ubiquitous oxidoreductase that participates in cellular defense and oxidative stress by controlling cellular free radicals and reactive oxygen species [25]. TXN is essential for the inflammatory response and is being investigated for its use in the treatment of many cancers [26]. TXN is an important part of the thiol-dependent antioxidant system, which can maintain the level of glutathione (GSH), inhibit the iron-dependent lipid peroxidation process, and play an important role in ferroptosis [27]. Nuclear factor of activated T cells 2 (NFATC2) belongs to a family of transcription factors that can be activated by calcium influx and is essential for T cell differentiation. In patients with asthma, NFATC2 is associated with peripheral eosinophilia [28]. NFATC2 has been shown to control inflammation and apoptosis [29, 30], but its role in sepsis has not been reported for now. GSTO1, a gene of the glutathione S-transferase family, is upregulated in a variety of highly aggressive cancer cells. A proteomic study showed that GSTO1 is mainly expressed in macrophages, widely involved in inflammatory and immune responses, and highly expressed in septic plasma, which is a potential research target for sepsis, but needs to be confirmed by more samples [31]. The above studies suggest that the proteins encoded by these five genes are abnormally expressed in inflammation-related diseases and are closely related to multiple programmed cell death patterns, indicating the potential diagnostic value of these five characteristic biomarkers in sepsis. Subsequently, the model we constructed confirmed the diagnostic validity of the five characteristic biomarkers for sepsis in the ROC curve.

MiRNAs are associated with the pathophysiological processes of many diseases [32]. Therefore, we constructed the TF-miRNA-hub network based on public datasets and published literature. We identified six TFs and 171 miRNAs as master regulators of the final gene regulatory network with the greatest connectivity to the five co-expressed genes associated with sepsis. Finally, we screened for drugs targeting the hub genes using DGIdb. Tasquinimod and Paquinimod are currently commonly used pharmacological inhibitors of S100A9, and it has been found that inhibiting S100A9 with Paquinimod can reduce sepsis induced mitochondrial dysfunction, oxidative stress, and immunosuppression through multiple pathways [33, 34]. Both PX-12 [35] and Gambogic acid [36] have been used in cancer research as inhibitors of TXN, but the effects of Gambogenic acid and Biotiny lated gambogic acid on TXN have not been reported. NFATC2 is closely related to asparaginase hypersensitivity [37], but the direct relationship between the two has not been clearly reported. Of course, the safety and reliability of these drugs will require further investigation in animal and clinical trials.

Based on bioinformatics technology and public databases, this study screened the key genes related to PCD in sepsis, in order to further find biomarkers that can achieve early diagnosis, predict therapeutic drugs with target genes as potential targets, and analyze the distribution of target genes in different immune cells using single-cell sequencing data sets. To explore the specific regulatory relationship of immune cells from single-cell sequencing combined with transcription factor expression. It has also been proved that sepsis affects the levels of S100A9, TXN and GSTO1 from the perspective of genetics, but the underlying mechanism is still unclear and needs to be further verified by basic experiments. This study provides a new method for revealing the regulatory mechanism of host response in sepsis, improving the diagnosis and treatment of patients with sepsis, and suggests that GSTO1 with bidirectional causal association may be an important marker in the progression of sepsis.

Despite these findings, our study has several limitations. First, we only extracted data from public databases, without animal experiments or clinical validation, and look forward to perfecting relevant experiments in further research. Second, the data used in this study were not from a Chinese population, but from a European population, and the results need to be further validated in a Chinese population. In addition, the sample size of GWAS studies in the Finnish database used for the study was small, and the number of SNPs screened using strict genome-wide thresholds was small. Therefore, the threshold criteria for screening instrumental variables were lowered in this study, which may lead to the influence of weak instrumental effects on the results, which needs to be verified in more comprehensive GWAS studies with larger sample sizes. Finally, GWAS data were relatively limited and MR Analysis could not be performed to assess whether there is a nonlinear relationship between sepsis and S100A9, TXN and GSTO1 levels.

## Conclusion

In this study, we used bioinformatics methods to identify diagnostic marker genes related to sepsis and PCD, and screened 7 drugs that could be used as candidate drugs for the treatment of sepsis. In addition, from the genetic perspective of Mendelian randomization analysis, GSTO1 with bidirectional causal association may be an important marker of sepsis. This study provides a workflow for predicting biomarkers and drug targets that can be widely applied to other diseases.

## Electronic supplementary material

Below is the link to the electronic supplementary material.

Supplementary Material 1

Supplementary Material 2

## Data Availability

Gene Expression Omnibus (GEO): http://www.ncbi.nlm.nih.gov/geo MSigDB (Molecular Signatures Database): http://software.broadinstitute.org/gsea/msigdb/index.jsp KEGG (Kyoto Encyclopedia of Genes and Genomes): https://www.kegg.jp/kegg/rest/keggapi.html FerrDb V2: http://www.zhounan.org/ferrdb/current/ GeneCards: https://www.genecards.org/ miRNet2.0: https://www.mirnet.ca/ DGIdb (Drug Gene Interaction database): https://www.dgidb.org/ IEU OpenGWAS: https://gwas.mrcieu.ac.uk/ FinnGen: https://www.finngen.fi/ PhenoScanner: http://www.phenoscanner.medschl.cam.ac.uk/.
