# Supplementary material for: Screening and analysis of programmed cell death related genes and targeted drugs in sepsis
Source: Hereditas. 2025 Mar 19;162:40. doi: 10.1186/s41065-025-00403-w (PMC11921706; doi:10.1186/s41065-025-00403-w)
Supplement: Supplementary file 1 — Supplementary Material 1 [file 41065_2025_403_MOESM1_ESM.docx]

Supplemental materials:

Eighty-eight cross-gene names of Venn analysis from PCD-related genes, WGCNA-related genes and DEGs:

GZMB, NAIP, ANXA1, EEF2K, IL32, PRF1, MPEG1, PARP1, SERPINB1, DNMT1, CHMP7, LY96, IRAK3, GSTO1, CLEC5A, HLA-DRB1, MMP9, CD27, F5, HGF, IL2RB, CCL5, CCR6, MAP3K14, STAT4, RETN, LCN2, HP, ADA2, CD8A, MMP8, BPI, PGLYRP1, FLOT1, GNLY, TXN, TP53I3, CD74, SGK1, PADI4, CTSD, TRIM28, AGFG1, EEF2, LEF1, PEBP1, LAMTOR5, DDIAS, BCL2A1, RASGRP1, BAG3, TCN1, PDCD4, LCK, S100A9, CD3E, CD247, CEACAM1, CD79A, MME, MS4A1, FYN, CD2, CD81, DPP4, CCR7, ETS1, NFATC2, CR1, WIPI1, SLC2A3, SLC2A14, POR, LPIN1, CAPG, GADD45A, FCMR, ITK, PRKCQ, G0S2, MTF1, ATIC, DRAM1, SH3GLB1, WDFY3, LTF, RUNX3, ANKRD36B.

Table Supplementary 1 is a separate document on 6 transcription factors and 171 miRNAs that target 5 genes (IRAK3, S100A9, TXN, NFATC2 and GSTO1).

Table S2. The instrumental variable information about S100A9

| SNP | EA | OA | P value | β | SE | Exposure |
| --- | --- | --- | --- | --- | --- | --- |
| rs10750532 | A | G | 1.67E-06 | -0.20786 | 0.04335 | S100A9 |
| rs112225971 | A | G | 2.02E-06 | 0.282044 | 0.059298 | S100A9 |
| rs116930480 | T | C | 4.41E-07 | -0.18416 | 0.036422 | S100A9 |
| rs12213817 | T | C | 2.49E-06 | 0.159375 | 0.033813 | S100A9 |
| rs140813586 | A | G | 2.93E-06 | -0.25304 | 0.05406 | S100A9 |
| rs150902100 | G | A | 1.03E-06 | -0.3149 | 0.064368 | S100A9 |
| rs2341674 | T | C | 1.9E-06 | -0.12257 | 0.025704 | S100A9 |
| rs2386663 | T | C | 5.63E-07 | -0.09743 | 0.019447 | S100A9 |
| rs3886106 | G | A | 2.08E-06 | 0.113526 | 0.023899 | S100A9 |
| rs671218 | C | T | 3.84E-06 | -0.09829 | 0.021254 | S100A9 |
| rs73120123 | T | C | 1.59E-06 | 0.272133 | 0.056636 | S100A9 |
| rs75514357 | A | G | 5.53E-07 | -0.20796 | 0.041484 | S100A9 |
| rs79514526 | G | A | 3.43E-06 | -0.24179 | 0.052016 | S100A9 |
| rs9808399 | G | A | 1.16E-07 | -0.19374 | 0.036504 | S100A9 |
| rs12503711 | C | T | 2.8E-06 | 0.0868 | 0.018506 | S100A9 |
| rs2588511 | G | C | 4.79E-06 | -0.08354 | 0.018245 | S100A9 |
| rs4937533 | C | T | 2.68E-06 | -0.1017 | 0.021642 | S100A9 |
| rs6804931 | C | T | 5.93E-07 | 0.090256 | 0.018053 | S100A9 |
| rs72606673 | G | A | 4.34E-06 | 0.111508 | 0.024245 | S100A9 |
| rs7544758 | T | C | 5.26E-07 | 0.096156 | 0.019144 | S100A9 |
| rs115479735 | T | C | 3.09E-06 | 0.6638 | 0.1423 | S100A9 |
| rs77076757 | A | G | 4.73E-06 | 0.309234 | 0.067501 | S100A9 |
| rs7838052 | A | G | 4.47E-06 | -0.11303 | 0.024609 | S100A9 |
| rs7935718 | C | T | 2.16E-06 | -0.0874 | 0.018428 | S100A9 |
| rs11625266 | C | T | 1.66E-06 | 0.1239 | 0.0259 | S100A9 |
| rs12970889 | C | T | 4.17E-06 | 0.2912 | 0.0632 | S100A9 |
| rs13276307 | C | T | 1.02E-06 | 0.1396 | 0.0286 | S100A9 |
| rs140843506 | G | A | 4.17E-06 | 0.4302 | 0.0935 | S100A9 |
| rs145720329 | T | G | 3.02E-06 | -0.4728 | 0.1012 | S100A9 |
| rs34436714 | C | A | 1.45E-95 | 0.5893 | 0.0284 | S100A9 |
| rs35431 | C | T | 3.47E-06 | -0.1289 | 0.0278 | S100A9 |
| rs4510697 | T | G | 7.59E-07 | -0.3764 | 0.0761 | S100A9 |
| rs4634868 | G | A | 9.12E-07 | 0.1453 | 0.0296 | S100A9 |
| rs537112422 | G | A | 2.34E-07 | 0.5253 | 0.1016 | S100A9 |
| rs55637274 | G | A | 4.9E-06 | -0.2842 | 0.0622 | S100A9 |
| rs6824171 | A | G | 4.79E-06 | 0.3616 | 0.0791 | S100A9 |
| rs7319037 | A | G | 2.69E-06 | 0.149 | 0.0318 | S100A9 |
| rs76111487 | A | T | 4.47E-06 | -0.4285 | 0.0934 | S100A9 |

Note: SNP: single nucleotide polymorphism, EA: effector allele, OA: non-effector allele, and SE: standard error of β.

Table S3. The instrumental variable information about TXN

| SNP | EA | OA | P value | β | SE | exposure |
| --- | --- | --- | --- | --- | --- | --- |
| rs115304750 | G | A | 4.57E-06 | -0.18444 | 0.040197 | TXN |
| rs11630690 | T | C | 4.47E-06 | 0.148463 | 0.032323 | TXN |
| rs148667088 | A | G | 4.57E-06 | 0.252844 | 0.055104 | TXN |
| rs181834694 | T | A | 4.91E-06 | 0.408108 | 0.089242 | TXN |
| rs1973376 | C | T | 2.73E-06 | 0.088844 | 0.018923 | TXN |
| rs3096694 | T | C | 9.65E-07 | 0.11761 | 0.023981 | TXN |
| rs4295631 | C | G | 1.15E-06 | -0.14378 | 0.029524 | TXN |
| rs58101364 | C | G | 1.34E-06 | -0.14435 | 0.029827 | TXN |
| rs72771835 | A | T | 4.76E-06 | -0.15814 | 0.03453 | TXN |
| rs76320753 | C | T | 1.15E-06 | -0.35007 | 0.0719 | TXN |
| rs77130185 | C | T | 2.03E-06 | -0.28452 | 0.059826 | TXN |
| rs7759301 | C | G | 3E-06 | -0.10044 | 0.02148 | TXN |
| rs8068839 | A | G | 5.96E-07 | -0.18992 | 0.037994 | TXN |
| rs11574452 | A | C | 2.67E-06 | -0.27445 | 0.058398 | TXN |
| rs116422947 | T | G | 2.81E-07 | 0.245619 | 0.047763 | TXN |
| rs12944509 | A | T | 2.16E-07 | 0.103733 | 0.019981 | TXN |
| rs140785326 | A | G | 1.84E-06 | -0.31594 | 0.066152 | TXN |
| rs1480699 | C | T | 3.24E-06 | -0.08764 | 0.018808 | TXN |
| rs17143628 | T | C | 4E-06 | 0.10012 | 0.021689 | TXN |
| rs234483 | C | T | 4.27E-06 | -0.09481 | 0.0206 | TXN |
| rs7564931 | G | A | 2.88E-07 | 0.110238 | 0.021457 | TXN |
| rs75697612 | G | A | 1.06E-06 | -0.25792 | 0.052795 | TXN |
| rs8180481 | C | T | 3.84E-06 | -0.08838 | 0.01911 | TXN |

Note: SNP: single nucleotide polymorphism, EA: effector allele, OA: non-effector allele, and SE: standard error of β.

Table S4. The instrumental variable information about GSTO1

| SNP | EA | OA | P value | β | SE | exposure |
| --- | --- | --- | --- | --- | --- | --- |
| rs10864296 | A | T | 2.28E-06 | 0.102087 | 0.021574 | GSTO1 |
| rs117697664 | G | A | 5.89E-13 | -0.27118 | 0.037555 | GSTO1 |
| rs12280390 | G | A | 1.3E-06 | 0.215624 | 0.044505 | GSTO1 |
| rs138816383 | G | A | 3.23E-06 | 0.33705 | 0.07232 | GSTO1 |
| rs145608462 | C | T | 1.25E-06 | -0.38563 | 0.079465 | GSTO1 |
| rs146125325 | G | A | 1.93E-06 | -0.29566 | 0.062042 | GSTO1 |
| rs2282326 | C | A | 3.7E-239 | -0.60333 | 0.017345 | GSTO1 |
| rs4842700 | A | G | 1.29E-06 | 0.115434 | 0.023813 | GSTO1 |
| rs56318108 | G | T | 1.12E-07 | 0.24468 | 0.046057 | GSTO1 |
| rs704 | G | A | 3.39E-09 | -0.10793 | 0.018227 | GSTO1 |
| rs72809840 | A | G | 1.84E-11 | -0.4116 | 0.061132 | GSTO1 |
| rs74193819 | C | T | 1.06E-07 | -0.21807 | 0.040967 | GSTO1 |
| rs75557043 | A | G | 1.6E-06 | 0.343372 | 0.071488 | GSTO1 |
| rs77630239 | T | G | 8.93E-07 | -0.34709 | 0.070552 | GSTO1 |
| rs7794237 | C | T | 4.62E-06 | -0.13416 | 0.029254 | GSTO1 |
| rs77949871 | A | C | 2.21E-06 | -0.29359 | 0.061957 | GSTO1 |
| rs9669571 | A | G | 3.87E-06 | 0.090878 | 0.019657 | GSTO1 |
| rs974799 | T | C | 4.6E-06 | 0.136447 | 0.029748 | GSTO1 |

Note: SNP: single nucleotide polymorphism, EA: effector allele, OA: non-effector allele, and SE: standard error of β.
