# Supplementary material for: Screening and analysis of programmed cell death related genes and targeted drugs in sepsis
Source: Hereditas. 2025 Mar 19;162:40. doi: 10.1186/s41065-025-00403-w (PMC11921706; doi:10.1186/s41065-025-00403-w)
Supplement: Supplementary file 2 — Supplementary Material 2 [file 41065_2025_403_MOESM2_ESM.docx]

| **ID** | **Target** | **TargetID** | **Experiment** | **Literature** |
| --- | --- | --- | --- | --- |
| CEBPA | S100A9 | 6280 | + | 9706399 |
| CEBPB | S100A9 | 6280 | + | 9706399 |
| GLI1 | S100A9 | 6280 | Unknown | 16880536 |
| HSF2 | TXN | 7295 | Unknown | 9374530 |
| JUN | TXN | 7295 | Unknown | 12414792 |
| PPARD | TXN | 7295 | + | 18048767 |
| hsa-let-7a-5p | GSTO1 | 9446 | HITS-CLIP, PAR-CLIP | 37986224 |
| hsa-let-7a-5p | IRAK3 | 11213 | HITS-CLIP | 24389009 |
| hsa-let-7a-5p | NFATC2 | 4773 | PAR-CLIP//HITS-CLIP | 22100165\|22291592\|23592263\|24668909\|24906430\|26061048\|27150721\|27292025 |
| hsa-let-7a-5p | TXN | 7295 | Chimeric fragments | 37986224 |
| hsa-let-7b-5p | GSTO1 | 9446 | HITS-CLIP, PAR-CLIP | 37986224 |
| hsa-let-7b-5p | IRAK3 | 11213 | Microarrays | 37986224 |
| hsa-let-7b-5p | NFATC2 | 4773 | PAR-CLIP//HITS-CLIP | 22100165\|22291592\|24906430\|27150721 |
| hsa-let-7b-5p | S100A9 | 6280 | PAR-CLIP | 27292025 |
| hsa-let-7b-5p | TXN | 7295 | Microarrays, Chimeric fragments | 37986224 |
| hsa-let-7c-5p | GSTO1 | 9446 | HITS-CLIP | 37986224 |
| hsa-let-7c-5p | NFATC2 | 4773 | PAR-CLIP//HITS-CLIP | 22291592\|26061048\|24906430\|27150721\|31101765 |
| hsa-let-7c-5p | S100A9 | 6280 | PAR-CLIP | 27292025 |
| hsa-let-7c-5p | TXN | 7295 | Chimeric fragments | 37986224 |
| hsa-let-7d-5p | GSTO1 | 9446 | PAR-CLIP | 23592263\|26701625\|27292025 |
| hsa-let-7d-5p | NFATC2 | 4773 | HITS-CLIP//PAR-CLIP | 27150721\|27292025 |
| hsa-let-7e-5p | GSTO1 | 9446 | HITS-CLIP, PAR-CLIP | 37986224 |
| hsa-let-7e-5p | NFATC2 | 4773 | PAR-CLIP//HITS-CLIP | 22100165\|24389009\|26061048\|24906430\|26701625\|27292025\|27150721 |
| hsa-let-7f-5p | GSTO1 | 9446 | HITS-CLIP, PAR-CLIP | 37986224 |
| hsa-let-7f-5p | NFATC2 | 4773 | PAR-CLIP//HITS-CLIP | 22012620\|22291592\|24906430\|27150721\|31101765 |
| hsa-let-7f-5p | TXN | 7295 | Chimeric fragments | 37986224 |
| hsa-miR-15a-5p | IRAK3 | 11213 | HITS-CLIP | 24389009 |
| hsa-miR-15a-5p | NFATC2 | 4773 | HITS-CLIP//PAR-CLIP | 22927820\|23313552\|24668909\|27150721\|26602609\|26962949\|26061048\|35269913 |
| hsa-miR-16-5p | GSTO1 | 9446 | pSILAC, Microarrays | 37986224 |
| hsa-miR-16-5p | IRAK3 | 11213 | CLASH | 23622248 |
| hsa-miR-16-5p | NFATC2 | 4773 | PAR-CLIP//HITS-CLIP | 24668909\|26061048\|27150721 |
| hsa-miR-16-5p | TXN | 7295 | Microarrays | 37986224 |
| hsa-miR-17-5p | NFATC2 | 4773 | PAR-CLIP//HITS-CLIP//R-Seq | 22100165\|22927820\|23313552\|24038734\|24668909\|27150721\|26061048\|26602609\|30670076 |
| hsa-miR-17-5p | TXN | 7295 | PAR-CLIP//HITS-CLIP | 37986224\|22100165\|22291592\|23313552\|24389009\|24668909\|26701625\|30455455\|27150721 |
| hsa-miR-18a-5p | NFATC2 | 4773 | PAR-CLIP//HITS-CLIP | 22100165\|23313552\|24038734\|24389009\|24668909\|24906430\|26061048\|27150721 |
| hsa-miR-18a-5p | S100A9 | 6280 | HITS-CLIP | 24906430 |
| hsa-miR-19a-3p | IRAK3 | 11213 | HITS-CLIP | 24389009 |
| hsa-miR-19a-3p | NFATC2 | 4773 | HITS-CLIP | 24906430\|27150721 |
| hsa-miR-19b-3p | IRAK3 | 11213 | HITS-CLIP | 24389009 |
| hsa-miR-19b-3p | NFATC2 | 4773 | HITS-CLIP | 27150721\|24906430\|34031238 |
| hsa-miR-20a-5p | GSTO1 | 9446 | Microarrays | 37986224 |
| hsa-miR-20a-5p | NFATC2 | 4773 | HITS-CLIP//PAR-CLIP | 24038734\|22100165\|22927820\|23313552\|23592263\|24668909\|26602609\|27150721 |
| hsa-miR-20a-5p | S100A9 | 6280 | Microarrays | 37986224 |
| hsa-miR-20a-5p | TXN | 7295 | PAR-CLIP//HITS-CLIP | 37986224\|22100165\|22291592\|24389009\|23313552\|24668909\|27150721\|26701625\|30455455 |
| hsa-miR-21-5p | TXN | 7295 | PAR-CLIP | 26701625 |
| hsa-miR-23a-3p | TXN | 7295 | HITS-CLIP | 24906430 |
| hsa-miR-24-3p | IRAK3 | 11213 | HITS-CLIP | 23824327 |
| hsa-miR-24-3p | NFATC2 | 4773 | HITS-CLIP | 37986224 |
| hsa-miR-25-3p | NFATC2 | 4773 | HITS-CLIP | 24906430 |
| hsa-miR-26a-5p | IRAK3 | 11213 | HITS-CLIP | 24389009 |
| hsa-miR-26a-5p | NFATC2 | 4773 | HITS-CLIP | 26061048\|27150721 |
| hsa-miR-26a-5p | TXN | 7295 | HITS-CLIP | 37986224\|24389009\|26061048\|30455455 |
| hsa-miR-26b-5p | IRAK3 | 11213 | HITS-CLIP | 24389009 |
| hsa-miR-26b-5p | NFATC2 | 4773 | HITS-CLIP | 27150721 |
| hsa-miR-26b-5p | TXN | 7295 | HITS-CLIP | 23313552\|24038734 |
| hsa-miR-27a-3p | GSTO1 | 9446 | HITS-CLIP | 37986224 |
| hsa-miR-27a-3p | IRAK3 | 11213 | Microarrays//HITS-CLIP | 37986224\|24389009\|23449803 |
| hsa-miR-27a-3p | NFATC2 | 4773 | PAR-CLIP | 24668909\|26701625 |
| hsa-miR-27a-3p | S100A9 | 6280 | HITS-CLIP//PAR-CLIP | 24906430\|27150721\|27292025 |
| hsa-miR-28-5p | NFATC2 | 4773 | HITS-CLIP | 24389009 |
| hsa-miR-29a-3p | NFATC2 | 4773 | HITS-CLIP | 26061048 |
| hsa-miR-30a-5p | NFATC2 | 4773 | HITS-CLIP | 27150721\|26061048 |
| hsa-miR-30a-3p | GSTO1 | 9446 | PAR-CLIP | 27292025 |
| hsa-miR-33a-5p | IRAK3 | 11213 | HITS-CLIP | 24389009 |
| hsa-miR-92a-3p | NFATC2 | 4773 | HITS-CLIP//qCLASH | 23313552\|24906430\|33956915 |
| hsa-miR-92a-3p | TXN | 7295 | PAR-CLIP//HITS-CLIP//qCLASH | 22100165\|24389009\|22291592\|22927820\|23313552\|26061048\|24906430\|27150721\|26701625\|30455455\|27292025\|33956915 |
| hsa-miR-93-5p | NFATC2 | 4773 | PAR-CLIP//HITS-CLIP | 22100165\|22927820\|23313552\|23592263\|24038734\|24668909\|26602609\|26061048\|27150721 |
| hsa-miR-93-5p | TXN | 7295 | PAR-CLIP, Chimeric fragments//PAR-CLIP//Chimeric fragments | 37986224\|22100165\|22291592\|24668909\|24857550 |
| hsa-miR-96-5p | S100A9 | 6280 | PAR-CLIP | 27292025 |
| hsa-miR-98-5p | GSTO1 | 9446 | HITS-CLIP, PAR-CLIP//PAR-CLIP | 37986224\|23592263\|26701625\|27292025 |
| hsa-miR-98-5p | NFATC2 | 4773 | HITS-CLIP | 27150721 |
| hsa-miR-101-3p | NFATC2 | 4773 | HITS-CLIP | 26061048\|24389009\|27150721 |
| hsa-miR-101-3p | S100A9 | 6280 | Microarrays | 37986224 |
| hsa-miR-29b-3p | GSTO1 | 9446 | PAR-CLIP//sR-Seq | 23592263\|33184237 |
| hsa-miR-29b-3p | S100A9 | 6280 | HITS-CLIP | 24389009 |
| hsa-miR-103a-3p | GSTO1 | 9446 | PAR-CLIP | 26701625 |
| hsa-miR-103a-3p | IRAK3 | 11213 | HITS-CLIP | 24389009 |
| hsa-miR-103a-3p | NFATC2 | 4773 | HITS-CLIP//PAR-CLIP | 22927820\|24668909\|27150721\|26962949 |
| hsa-miR-103a-3p | TXN | 7295 | PAR-CLIP | 26701625 |
| hsa-miR-106a-5p | NFATC2 | 4773 | HITS-CLIP//PAR-CLIP | 22927820\|23313552\|24668909\|34031238 |
| hsa-miR-106a-5p | TXN | 7295 | HITS-CLIP, PAR-CLIP//PAR-CLIP//HITS-CLIP | 37986224\|22100165\|22291592\|23313552\|24389009\|24668909\|30455455 |
| hsa-miR-107 | IRAK3 | 11213 | HITS-CLIP | 24389009 |
| hsa-miR-107 | NFATC2 | 4773 | HITS-CLIP//PAR-CLIP | 22927820\|24668909\|27150721\|26061048\|26962949 |
| hsa-miR-192-5p | NFATC2 | 4773 | HITS-CLIP | 37986224 |
| hsa-miR-196a-5p | GSTO1 | 9446 | PAR-CLIP | 23592263\|26701625\|27292025 |
| hsa-miR-196a-5p | IRAK3 | 11213 | HITS-CLIP | 24038734 |
| hsa-miR-196a-5p | NFATC2 | 4773 | PAR-CLIP//HITS-CLIP | 20371350\|22100165\|22291592\|22927820\|23313552\|24668909\|28925592\|27150721\|31101765 |
| hsa-miR-196a-5p | S100A9 | 6280 | Luciferase reporter assay//qRT-PCR//Other | 19342367\|20026422 |
| hsa-miR-196a-5p | TXN | 7295 | PAR-CLIP | 27292025 |
| hsa-miR-197-3p | GSTO1 | 9446 | CLASH | 23622248 |
| hsa-miR-197-3p | TXN | 7295 | HITS-CLIP | 37986224 |
| hsa-miR-199a-3p | NFATC2 | 4773 | HITS-CLIP | 26061048 |
| hsa-miR-129-5p | NFATC2 | 4773 | HITS-CLIP | 27418678 |
| hsa-miR-148a-3p | NFATC2 | 4773 | R-Seq | 36357926 |
| hsa-miR-30d-5p | NFATC2 | 4773 | HITS-CLIP | 26061048 |
| hsa-miR-139-5p | IRAK3 | 11213 | HITS-CLIP | 24389009 |
| hsa-miR-139-5p | NFATC2 | 4773 | HITS-CLIP | 24389009\|26061048 |
| hsa-miR-139-5p | TXN | 7295 | HITS-CLIP | 24389009 |
| hsa-miR-7-5p | NFATC2 | 4773 | HITS-CLIP | 26061048\|27150721 |
| hsa-miR-10a-5p | NFATC2 | 4773 | PAR-CLIP | 27292025 |
| hsa-miR-34a-5p | GSTO1 | 9446 | PAR-CLIP | 23592263\|26701625 |
| hsa-miR-34a-5p | IRAK3 | 11213 | HITS-CLIP | 24389009\|34031238 |
| hsa-miR-34a-5p | NFATC2 | 4773 | PAR-CLIP//HITS-CLIP//R-Seq | 22291592\|24906430\|26061048\|26602609\|26701625\|27150721\|27292025\|30670076 |
| hsa-miR-34a-5p | TXN | 7295 | HITS-CLIP | 24038734\|24389009\|24906430 |
| hsa-miR-181a-5p | NFATC2 | 4773 | HITS-CLIP | 22927820\|23313552\|26061048\|24389009\|26602609\|26962949\|27150721 |
| hsa-miR-181a-5p | TXN | 7295 | HITS-CLIP | 23313552\|24038734\|24906430\|26061048\|27150721\|27418678 |
| hsa-miR-181b-5p | NFATC2 | 4773 | HITS-CLIP | 24389009\|26061048\|26962949\|27150721 |
| hsa-miR-182-5p | GSTO1 | 9446 | Biotin-Microarrays//PAR-CLIP | 37986224\|26701625\|23249749 |
| hsa-miR-182-5p | IRAK3 | 11213 | PAR-CLIP | 22291592 |
| hsa-miR-182-5p | NFATC2 | 4773 | PAR-CLIP | 22291592 |
| hsa-miR-182-5p | S100A9 | 6280 | PAR-CLIP | 27292025 |
| hsa-miR-182-5p | TXN | 7295 | Biotin-Microarrays//PAR-CLIP//qCLASH | 37986224\|20371350\|22100165\|23592263\|24668909\|26701625\|34914716\|27292025\|23249749 |
| hsa-miR-182-3p | S100A9 | 6280 | PAR-CLIP | 27292025 |
| hsa-miR-183-5p | NFATC2 | 4773 | PAR-CLIP | 26701625 |
| hsa-miR-183-5p | TXN | 7295 | PAR-CLIP//HITS-CLIP | 22100165\|23313552\|23592263\|24668909\|26701625\|24906430\|26962949\|27150721\|26061048 |
| hsa-miR-203a-3p | NFATC2 | 4773 | PAR-CLIP | 26701625 |
| hsa-miR-210-3p | NFATC2 | 4773 | PAR-CLIP//HITS-CLIP | 22100165\|24389009\|24668909 |
| hsa-miR-214-3p | GSTO1 | 9446 | Microarrays | 37986224\|23100276 |
| hsa-miR-218-5p | S100A9 | 6280 | PAR-CLIP | 27292025 |
| hsa-miR-221-3p | NFATC2 | 4773 | HITS-CLIP | 22927820\|23313552\|26061048\|24389009\|26602609\|27150721 |
| hsa-miR-221-3p | TXN | 7295 | HITS-CLIP | 23313552\|24038734\|26061048 |
| hsa-miR-222-3p | NFATC2 | 4773 | HITS-CLIP | 26061048\|27150721 |
| hsa-miR-222-3p | TXN | 7295 | CLASH | 23622248 |
| hsa-miR-224-5p | NFATC2 | 4773 | Microarrays | 37986224 |
| hsa-miR-200b-3p | NFATC2 | 4773 | PAR-CLIP | 22012620\|22291592\|28925592 |
| hsa-let-7g-5p | GSTO1 | 9446 | HITS-CLIP, PAR-CLIP | 37986224 |
| hsa-let-7g-5p | IRAK3 | 11213 | HITS-CLIP | 24389009 |
| hsa-let-7g-5p | NFATC2 | 4773 | PAR-CLIP//HITS-CLIP | 20371350\|22100165\|22291592\|23313552\|24668909\|24906430\|26061048\|26701625\|27150721\|31101765 |
| hsa-let-7g-5p | TXN | 7295 | Chimeric fragments | 37986224 |
| hsa-let-7i-5p | GSTO1 | 9446 | HITS-CLIP, PAR-CLIP | 37986224 |
| hsa-let-7i-5p | IRAK3 | 11213 | HITS-CLIP | 24389009 |
| hsa-let-7i-5p | NFATC2 | 4773 | PAR-CLIP//HITS-CLIP | 20371350\|22100165\|22291592\|23592263\|24668909\|24906430\|28925592\|27150721\|31101765 |
| hsa-let-7i-5p | TXN | 7295 | Chimeric fragments | 37986224 |
| hsa-miR-1-3p | GSTO1 | 9446 | Proteomics//pSILAC | 18668040 |
| hsa-miR-1-3p | TXN | 7295 | AGO-IP, RPF-Seq, RNA-Seq, Microarrays//HITS-CLIP//R-Seq//Microarrays//RPF-Seq//AGO-IP | 37986224\|24389009\|24906430\|25263593\|18668040\|18461144 |
| hsa-miR-15b-5p | NFATC2 | 4773 | HITS-CLIP//PAR-CLIP | 22927820\|26061048\|24668909\|27150721\|26962949 |
| hsa-miR-23b-3p | GSTO1 | 9446 | RNA-Seq | 37986224 |
| hsa-miR-23b-3p | TXN | 7295 | RNA-Seq//R-Seq | 37986224\|23580553 |
| hsa-miR-27b-3p | NFATC2 | 4773 | PAR-CLIP | 26701625 |
| hsa-miR-27b-3p | S100A9 | 6280 | HITS-CLIP//PAR-CLIP | 24906430\|27292025\|27150721 |
| hsa-miR-122-5p | IRAK3 | 11213 | Biotin-Microarrays | 37986224\|21937511 |
| hsa-miR-124-3p | GSTO1 | 9446 | Other | 37986224 |
| hsa-miR-124-3p | IRAK3 | 11213 | Microarray//Microarrays//R-Seq | 18668037\|30292704 |
| hsa-miR-124-3p | NFATC2 | 4773 | HITS-CLIP | 24389009 |
| hsa-miR-124-3p | TXN | 7295 | Other, Microarrays | 37986224 |
| hsa-miR-128-3p | NFATC2 | 4773 | Microarrays | 37986224\|21811625 |
| hsa-miR-128-3p | S100A9 | 6280 | PAR-CLIP | 27292025 |
| hsa-miR-128-3p | TXN | 7295 | R-Seq | 36357926 |
| hsa-miR-135a-5p | GSTO1 | 9446 | HITS-CLIP | 37986224 |
| hsa-miR-137 | GSTO1 | 9446 | HITS-CLIP | 37986224 |
| hsa-miR-138-5p | TXN | 7295 | HITS-CLIP | 24389009 |
| hsa-miR-142-5p | NFATC2 | 4773 | PAR-CLIP | 22100165\|23592263\|24668909 |
| hsa-miR-142-3p | NFATC2 | 4773 | PAR-CLIP | 22100165\|23592263\|24668909 |
| hsa-miR-191-5p | GSTO1 | 9446 | Microarrays | 37986224 |
| hsa-miR-191-5p | IRAK3 | 11213 | HITS-CLIP | 24038734 |
| hsa-miR-191-5p | NFATC2 | 4773 | Microarrays | 37986224\|20924108 |
| hsa-miR-191-5p | TXN | 7295 | HITS-CLIP | 23313552\|24038734\|24906430\|26061048\|27150721\|27418678 |
| hsa-miR-9-5p | NFATC2 | 4773 | PAR-CLIP//R-Seq | 22291592\|30670076 |
| hsa-miR-9-3p | NFATC2 | 4773 | PAR-CLIP//R-Seq | 37986224\|30670076 |
| hsa-miR-126-3p | S100A9 | 6280 | Microarrays | 37986224\|27070706 |
| hsa-miR-126-3p | TXN | 7295 | Microarrays | 37986224 |
| hsa-miR-146a-5p | NFATC2 | 4773 | HITS-CLIP | 24389009\|26061048 |
| hsa-miR-146a-5p | TXN | 7295 | HITS-CLIP | 23313552 |
| hsa-miR-149-5p | GSTO1 | 9446 | HITS-CLIP | 37986224 |
| hsa-miR-149-5p | TXN | 7295 | CLASH | 23622248 |
| hsa-miR-184 | NFATC2 | 4773 | Luciferase reporter assay//qRT-PCR//Western blot//Other | 19286996 |
| hsa-miR-185-5p | NFATC2 | 4773 | PAR-CLIP | 22291592 |
| hsa-miR-194-5p | NFATC2 | 4773 | PAR-CLIP | 24668909 |
| hsa-miR-195-5p | NFATC2 | 4773 | HITS-CLIP | 22927820\|27150721 |
| hsa-miR-206 | NFATC2 | 4773 | R-Seq | 30670076 |
| hsa-miR-206 | TXN | 7295 | HITS-CLIP | 27418678 |
| hsa-miR-200c-3p | NFATC2 | 4773 | Microarrays//PAR-CLIP//R-Seq | 37986224\|22100165\|22291592\|28925592\|30670076 |
| hsa-miR-200c-3p | TXN | 7295 | qCLASH | 33956915 |
| hsa-miR-155-5p | NFATC2 | 4773 | Microarrays//PAR-CLIP | 37986224\|22100165\|22291592 |
| hsa-miR-155-5p | TXN | 7295 | PAR-CLIP | 37986224 |
| hsa-miR-106b-5p | NFATC2 | 4773 | PAR-CLIP//HITS-CLIP | 22100165\|22927820\|23313552\|24038734\|26602609\|24668909\|27150721 |
| hsa-miR-106b-5p | TXN | 7295 | PAR-CLIP//HITS-CLIP | 37986224\|22100165\|22291592\|23313552\|24389009\|24668909\|30455455\|27150721 |
| hsa-miR-29c-3p | S100A9 | 6280 | HITS-CLIP | 24389009 |
| hsa-miR-301a-3p | NFATC2 | 4773 | HITS-CLIP | 27150721 |
| hsa-miR-301a-3p | TXN | 7295 | PAR-CLIP | 26701625 |
| hsa-miR-99b-5p | TXN | 7295 | PAR-CLIP | 27292025 |
| hsa-miR-296-5p | GSTO1 | 9446 | HITS-CLIP | 37986224 |
| hsa-miR-130b-3p | NFATC2 | 4773 | HITS-CLIP | 24906430 |
| hsa-miR-30e-3p | GSTO1 | 9446 | PAR-CLIP | 27292025 |
| hsa-miR-374a-5p | NFATC2 | 4773 | HITS-CLIP | 27150721 |
| hsa-miR-374a-5p | TXN | 7295 | Chimeric fragments | 37986224\|24857550 |
| hsa-miR-330-3p | S100A9 | 6280 | Microarrays | 37986224\|26967895 |
| hsa-miR-326 | NFATC2 | 4773 | PAR-CLIP | 27292025 |
| hsa-miR-151a-3p | TXN | 7295 | HITS-CLIP | 24389009\|26061048 |
| hsa-miR-135b-5p | GSTO1 | 9446 | HITS-CLIP | 37986224 |
| hsa-miR-135b-5p | IRAK3 | 11213 | qCLASH | 33956915 |
| hsa-miR-331-3p | S100A9 | 6280 | PAR-CLIP | 27292025 |
| hsa-miR-324-5p | NFATC2 | 4773 | HITS-CLIP | 27150721 |
| hsa-miR-324-3p | NFATC2 | 4773 | PAR-CLIP | 26701625 |
| hsa-miR-338-3p | NFATC2 | 4773 | PAR-CLIP | 22291592 |
| hsa-miR-338-3p | S100A9 | 6280 | PAR-CLIP | 27292025 |
| hsa-miR-133b | NFATC2 | 4773 | R-Seq | 30670076 |
| hsa-miR-345-5p | GSTO1 | 9446 | HITS-CLIP | 37986224 |
| hsa-miR-196b-5p | NFATC2 | 4773 | PAR-CLIP//HITS-CLIP | 20371350\|22100165\|23313552\|27292025\|28925592 |
| hsa-miR-423-3p | NFATC2 | 4773 | HITS-CLIP | 22927820 |
| hsa-miR-424-5p | NFATC2 | 4773 | HITS-CLIP//PAR-CLIP | 22927820\|26061048\|24668909\|27150721\|26962949\|35269913 |
| hsa-miR-18b-5p | NFATC2 | 4773 | HITS-CLIP//PAR-CLIP | 23313552\|24389009\|24668909 |
| hsa-miR-20b-5p | NFATC2 | 4773 | HITS-CLIP//PAR-CLIP | 22927820\|23313552\|24668909\|27292025 |
| hsa-miR-429 | NFATC2 | 4773 | PAR-CLIP | 22012620\|28925592 |
| hsa-miR-429 | S100A9 | 6280 | Microarrays | 37986224 |
| hsa-miR-450a-5p | IRAK3 | 11213 | R-Seq | 31101765 |
| hsa-miR-200a-5p | GSTO1 | 9446 | PAR-CLIP | 26701625 |
| hsa-miR-200a-5p | NFATC2 | 4773 | PAR-CLIP | 26701625 |
| hsa-miR-409-5p | IRAK3 | 11213 | HITS-CLIP | 24389009 |
| hsa-miR-409-3p | NFATC2 | 4773 | HITS-CLIP | 24389009 |
| hsa-miR-409-3p | TXN | 7295 | HITS-CLIP | 37986224\|24389009 |
| hsa-miR-484 | GSTO1 | 9446 | HITS-CLIP | 37986224 |
| hsa-miR-489-3p | NFATC2 | 4773 | HITS-CLIP | 27418678 |
| hsa-miR-146b-5p | NFATC2 | 4773 | HITS-CLIP | 24389009\|26061048 |
| hsa-miR-496 | NFATC2 | 4773 | HITS-CLIP | 23824327 |
| hsa-miR-181d-5p | NFATC2 | 4773 | HITS-CLIP | 23313552\|26061048\|24389009\|26602609\|26962949\|27150721 |
| hsa-miR-520f-3p | IRAK3 | 11213 | Microarrays | 37986224 |
| hsa-miR-526b-5p | NFATC2 | 4773 | PAR-CLIP | 22100165 |
| hsa-miR-525-3p | TXN | 7295 | 2D-DIGE, Luciferase Reporter Assay//2D-DIGE//Luciferase Reporter Assay | 37986224\|24147004 |
| hsa-miR-500a-3p | NFATC2 | 4773 | PAR-CLIP | 26701625 |
| hsa-miR-503-5p | NFATC2 | 4773 | HITS-CLIP | 27150721 |
| hsa-miR-505-3p | TXN | 7295 | HITS-CLIP//PAR-CLIP | 23313552\|27292025 |
| hsa-miR-514a-3p | NFATC2 | 4773 | HITS-CLIP | 27418678 |
| hsa-miR-92b-3p | NFATC2 | 4773 | HITS-CLIP | 24906430 |
| hsa-miR-92b-3p | TXN | 7295 | PAR-CLIP//HITS-CLIP | 20371350\|22100165\|22291592\|22927820\|23313552\|24389009\|26061048\|24668909\|24906430\|27150721\|26701625\|30455455 |
| hsa-miR-588 | S100A9 | 6280 | HITS-CLIP | 19536157 |
| hsa-miR-589-3p | GSTO1 | 9446 | HITS-CLIP | 37986224 |
| hsa-miR-593-5p | GSTO1 | 9446 | HITS-CLIP | 37986224 |
| hsa-miR-598-3p | NFATC2 | 4773 | PAR-CLIP | 22291592 |
| hsa-miR-603 | GSTO1 | 9446 | RNA-Seq | 37986224 |
| hsa-miR-603 | NFATC2 | 4773 | HITS-CLIP | 23824327 |
| hsa-miR-603 | TXN | 7295 | RNA-Seq | 37986224 |
| hsa-miR-33b-5p | IRAK3 | 11213 | HITS-CLIP | 24389009 |
| hsa-miR-652-3p | TXN | 7295 | HITS-CLIP//PAR-CLIP | 22927820\|23313552\|24389009\|26701625\|24906430\|27150721\|26061048\|26962949 |
| hsa-miR-671-5p | S100A9 | 6280 | Microarrays | 37986224 |
| hsa-miR-767-5p | TXN | 7295 | PAR-CLIP | 27292025 |
| hsa-miR-454-3p | NFATC2 | 4773 | HITS-CLIP | 27150721 |
| hsa-miR-769-5p | GSTO1 | 9446 | PAR-CLIP | 24668909 |
| hsa-miR-769-5p | NFATC2 | 4773 | PAR-CLIP | 27292025 |
| hsa-let-7f-1-3p | TXN | 7295 | HITS-CLIP | 23313552 |
| hsa-miR-15a-3p | NFATC2 | 4773 | PAR-CLIP | 22291592 |
| hsa-miR-16-1-3p | IRAK3 | 11213 | PAR-CLIP | 23592263 |
| hsa-miR-21-3p | TXN | 7295 | PAR-CLIP | 27292025 |
| hsa-miR-25-5p | NFATC2 | 4773 | PAR-CLIP | 26701625 |
| hsa-miR-29a-5p | NFATC2 | 4773 | PAR-CLIP | 22291592 |
| hsa-miR-33a-3p | TXN | 7295 | HITS-CLIP | 37986224 |
| hsa-miR-100-3p | GSTO1 | 9446 | PAR-CLIP | 26701625 |
| hsa-miR-105-3p | IRAK3 | 11213 | HITS-CLIP | 24389009 |
| hsa-miR-30c-2-3p | IRAK3 | 11213 | HITS-CLIP//PAR-CLIP | 23446348\|21572407\|20371350\|23824327 |
| hsa-miR-30d-3p | GSTO1 | 9446 | PAR-CLIP | 27292025 |
| hsa-miR-10a-3p | NFATC2 | 4773 | HITS-CLIP | 23824327\|27418678 |
| hsa-miR-181c-3p | NFATC2 | 4773 | HITS-CLIP | 27150721 |
| hsa-miR-199b-3p | NFATC2 | 4773 | HITS-CLIP | 26061048 |
| hsa-miR-221-5p | S100A9 | 6280 | PAR-CLIP | 27292025 |
| hsa-miR-30b-3p | IRAK3 | 11213 | HITS-CLIP//PAR-CLIP | 23446348\|21572407\|20371350\|23824327 |
| hsa-miR-30b-3p | S100A9 | 6280 | HITS-CLIP | 24906430 |
| hsa-miR-30b-3p | TXN | 7295 | HITS-CLIP | 24906430 |
| hsa-miR-125b-1-3p | TXN | 7295 | PAR-CLIP | 23592263\|26701625 |
| hsa-miR-132-5p | S100A9 | 6280 | HITS-CLIP | 19536157 |
| hsa-miR-141-5p | GSTO1 | 9446 | PAR-CLIP | 26701625 |
| hsa-miR-141-5p | NFATC2 | 4773 | PAR-CLIP | 26701625 |
| hsa-miR-144-5p | TXN | 7295 | HITS-CLIP | 23313552\|27418678 |
| hsa-miR-125a-3p | NFATC2 | 4773 | PAR-CLIP//HITS-CLIP | 22100165\|26061048 |
| hsa-miR-129-2-3p | NFATC2 | 4773 | Microarrays | 37986224\|22684256 |
| hsa-miR-149-3p | IRAK3 | 11213 | HITS-CLIP//PAR-CLIP | 23446348\|21572407\|20371350\|23824327 |
| hsa-miR-186-3p | GSTO1 | 9446 | HITS-CLIP | 37986224 |
| hsa-miR-195-3p | NFATC2 | 4773 | HITS-CLIP | 27150721 |
| hsa-miR-155-3p | IRAK3 | 11213 | Luciferase reporter assay//Microarray//qRT-PCR//Western blot | 20852130 |
| hsa-miR-29c-5p | TXN | 7295 | HITS-CLIP | 37986224 |
| hsa-miR-30c-1-3p | IRAK3 | 11213 | HITS-CLIP//PAR-CLIP | 23446348\|21572407\|20371350\|23824327 |
| hsa-miR-340-5p | TXN | 7295 | R-Seq | 34181735 |
| hsa-miR-331-5p | GSTO1 | 9446 | PAR-CLIP | 23592263 |
| hsa-miR-335-3p | GSTO1 | 9446 | PAR-CLIP//sR-Seq | 23592263\|33184237 |
| hsa-miR-335-3p | TXN | 7295 | PAR-CLIP//sR-Seq | 27292025\|33184237 |
| hsa-miR-423-5p | NFATC2 | 4773 | PAR-CLIP//HITS-CLIP | 26701625\|26061048\|27292025 |
| hsa-miR-423-5p | TXN | 7295 | HITS-CLIP//PAR-CLIP | 26061048\|27292025 |
| hsa-miR-20b-3p | NFATC2 | 4773 | HITS-CLIP | 23824327 |
| hsa-miR-193b-5p | GSTO1 | 9446 | PAR-CLIP | 27292025 |
| hsa-miR-193b-5p | TXN | 7295 | PAR-CLIP | 22100165 |
| hsa-miR-497-3p | GSTO1 | 9446 | HITS-CLIP | 37986224 |
| hsa-miR-92b-5p | TXN | 7295 | PAR-CLIP | 22100165 |
| hsa-miR-574-5p | GSTO1 | 9446 | R-Seq | 31562301 |
| hsa-miR-574-5p | S100A9 | 6280 | R-Seq | 31562301 |
| hsa-miR-548c-5p | TXN | 7295 | PAR-CLIP | 37986224 |
| hsa-miR-628-5p | GSTO1 | 9446 | HITS-CLIP | 37986224 |
| hsa-miR-629-5p | NFATC2 | 4773 | PAR-CLIP | 22100165\|24668909 |
| hsa-miR-629-5p | TXN | 7295 | PAR-CLIP//HITS-CLIP | 22100165\|26061048\|24906430\|26962949\|27150721 |
| hsa-miR-548d-5p | TXN | 7295 | PAR-CLIP | 37986224 |
| hsa-miR-450b-5p | TXN | 7295 | HITS-CLIP | 23313552 |
| hsa-miR-874-3p | GSTO1 | 9446 | HITS-CLIP | 37986224 |
| hsa-miR-876-5p | IRAK3 | 11213 | PAR-CLIP | 23592263 |
| hsa-miR-876-3p | IRAK3 | 11213 | HITS-CLIP | 24389009 |
| hsa-miR-876-3p | TXN | 7295 | HITS-CLIP | 24389009\|30455455 |
| hsa-miR-877-5p | TXN | 7295 | PAR-CLIP | 22100165\|23592263 |
| hsa-miR-374b-5p | NFATC2 | 4773 | PAR-CLIP//HITS-CLIP | 24668909\|27150721 |
| hsa-miR-374b-5p | TXN | 7295 | Chimeric fragments | 37986224\|24857550 |
| hsa-miR-301b-3p | NFATC2 | 4773 | HITS-CLIP | 27150721 |
| hsa-miR-940 | IRAK3 | 11213 | HITS-CLIP//PAR-CLIP | 23446348\|21572407\|20371350\|23824327 |
| hsa-miR-941 | IRAK3 | 11213 | Microarrays | 37986224 |
| hsa-miR-522-5p | NFATC2 | 4773 | IMPACT-Seq | 37986224 |
| hsa-miR-1226-3p | TXN | 7295 | HITS-CLIP | 37986224 |
| hsa-miR-320b | IRAK3 | 11213 | HITS-CLIP | 24389009 |
| hsa-miR-320b | NFATC2 | 4773 | PAR-CLIP | 26701625 |
| hsa-miR-320b | TXN | 7295 | HITS-CLIP | 24906430 |
| hsa-miR-320c | IRAK3 | 11213 | HITS-CLIP | 24389009 |
| hsa-miR-320c | TXN | 7295 | HITS-CLIP | 24389009 |
| hsa-miR-1296-5p | S100A9 | 6280 | PAR-CLIP | 27292025 |
| hsa-miR-1271-5p | TXN | 7295 | R-Seq | 32325711 |
| hsa-miR-1301-3p | TXN | 7295 | CLASH | 23622248 |
| hsa-miR-1183 | NFATC2 | 4773 | HITS-CLIP | 23824327 |
| hsa-miR-1204 | S100A9 | 6280 | HITS-CLIP | 19536157 |
| hsa-miR-1208 | NFATC2 | 4773 | HITS-CLIP | 23824327\|27418678 |
| hsa-miR-1287-5p | NFATC2 | 4773 | HITS-CLIP | 24389009\|27150721 |
| hsa-miR-548f-3p | TXN | 7295 | HITS-CLIP | 37986224 |
| hsa-miR-1270 | NFATC2 | 4773 | qRT-PCR | 34150008 |
| hsa-miR-1275 | GSTO1 | 9446 | PAR-CLIP | 27292025 |
| hsa-miR-1277-3p | TXN | 7295 | HITS-CLIP//PAR-CLIP | 37986224\|20371350 |
| hsa-miR-320d | NFATC2 | 4773 | PAR-CLIP | 22100165 |
| hsa-miR-1910-5p | S100A9 | 6280 | PAR-CLIP | 27292025 |
| hsa-let-7a-2-3p | NFATC2 | 4773 | HITS-CLIP | 26061048 |
| hsa-miR-3121-3p | TXN | 7295 | PAR-CLIP | 22100165 |
| hsa-miR-3122 | IRAK3 | 11213 | HITS-CLIP//PAR-CLIP | 23446348\|21572407\|20371350\|23824327 |
| hsa-miR-466 | NFATC2 | 4773 | HITS-CLIP | 23824327 |
| hsa-miR-3157-5p | NFATC2 | 4773 | HITS-CLIP | 23824327\|27418678 |
| hsa-miR-3178 | IRAK3 | 11213 | HITS-CLIP | 23824327 |
| hsa-miR-548w | TXN | 7295 | PAR-CLIP | 37986224 |
| hsa-miR-3184-5p | TXN | 7295 | PAR-CLIP | 27292025 |
| hsa-miR-514b-3p | NFATC2 | 4773 | HITS-CLIP | 27418678 |
| hsa-miR-4310 | GSTO1 | 9446 | PAR-CLIP | 27292025 |
| hsa-miR-4310 | NFATC2 | 4773 | PAR-CLIP | 22100165 |
| hsa-miR-4260 | GSTO1 | 9446 | PAR-CLIP | 27292025 |
| hsa-miR-4252 | S100A9 | 6280 | HITS-CLIP | 19536157 |
| hsa-miR-4284 | IRAK3 | 11213 | HITS-CLIP | 23824327 |
| hsa-miR-4291 | GSTO1 | 9446 | PAR-CLIP | 27292025 |
| hsa-miR-3652 | TXN | 7295 | PAR-CLIP | 27292025 |
| hsa-miR-3689a-3p | IRAK3 | 11213 | HITS-CLIP//PAR-CLIP | 23446348\|21572407\|20371350\|23824327 |
| hsa-miR-3691-5p | NFATC2 | 4773 | PAR-CLIP | 22100165\|24668909 |
| hsa-miR-3689b-3p | IRAK3 | 11213 | HITS-CLIP//PAR-CLIP | 23446348\|21572407\|20371350\|23824327 |
| hsa-miR-3913-5p | IRAK3 | 11213 | HITS-CLIP//PAR-CLIP | 23446348\|21572407\|20371350\|23824327 |
| hsa-miR-3934-5p | NFATC2 | 4773 | PAR-CLIP | 22100165 |
| hsa-miR-3934-5p | TXN | 7295 | PAR-CLIP | 22100165 |
| hsa-miR-3937 | IRAK3 | 11213 | HITS-CLIP//PAR-CLIP | 23446348\|21572407\|20371350\|23824327 |
| hsa-miR-3941 | NFATC2 | 4773 | HITS-CLIP | 23824327 |
| hsa-miR-378g | TXN | 7295 | PAR-CLIP | 22100165 |
| hsa-miR-4459 | NFATC2 | 4773 | HITS-CLIP | 23824327 |
| hsa-miR-3689c | IRAK3 | 11213 | HITS-CLIP//PAR-CLIP | 23446348\|21572407\|20371350\|23824327 |
| hsa-miR-4486 | IRAK3 | 11213 | HITS-CLIP | 23824327 |
| hsa-miR-4493 | IRAK3 | 11213 | HITS-CLIP | 23824327 |
| hsa-miR-4505 | IRAK3 | 11213 | HITS-CLIP | 23824327 |
| hsa-miR-4508 | IRAK3 | 11213 | HITS-CLIP | 23824327 |
| hsa-miR-4512 | NFATC2 | 4773 | HITS-CLIP | 23824327\|27418678 |
| hsa-miR-4514 | IRAK3 | 11213 | HITS-CLIP//PAR-CLIP | 23446348\|21572407\|20371350\|23824327 |
| hsa-miR-3940-5p | TXN | 7295 | PAR-CLIP | 22100165 |
| hsa-miR-4645-5p | IRAK3 | 11213 | HITS-CLIP//PAR-CLIP | 23446348\|21572407\|20371350\|23824327 |
| hsa-miR-4662a-5p | TXN | 7295 | PAR-CLIP | 22100165 |
| hsa-miR-4665-5p | GSTO1 | 9446 | PAR-CLIP | 27292025 |
| hsa-miR-4672 | NFATC2 | 4773 | HITS-CLIP | 23824327 |
| hsa-miR-4673 | IRAK3 | 11213 | HITS-CLIP//PAR-CLIP | 23446348\|21572407\|20371350\|23824327 |
| hsa-miR-4677-3p | GSTO1 | 9446 | PAR-CLIP | 26701625 |
| hsa-miR-4679 | S100A9 | 6280 | HITS-CLIP | 19536157 |
| hsa-miR-1343-3p | S100A9 | 6280 | RNA-Seq | 37986224 |
| hsa-miR-1343-3p | TXN | 7295 | RNA-Seq | 37986224 |
| hsa-miR-4692 | IRAK3 | 11213 | HITS-CLIP//PAR-CLIP | 23446348\|21572407\|20371350\|23824327 |
| hsa-miR-4700-3p | NFATC2 | 4773 | HITS-CLIP | 23824327 |
| hsa-miR-4701-5p | S100A9 | 6280 | HITS-CLIP | 19536157 |
| hsa-miR-451b | NFATC2 | 4773 | PAR-CLIP | 22100165 |
| hsa-miR-4728-5p | IRAK3 | 11213 | HITS-CLIP//PAR-CLIP | 23446348\|21572407\|20371350\|23824327 |
| hsa-miR-4738-3p | GSTO1 | 9446 | HITS-CLIP | 37986224 |
| hsa-miR-4755-3p | IRAK3 | 11213 | HITS-CLIP//PAR-CLIP | 23446348\|21572407\|20371350\|23824327 |
| hsa-miR-5571-5p | NFATC2 | 4773 | PAR-CLIP | 22100165 |
| hsa-miR-5572 | GSTO1 | 9446 | PAR-CLIP | 27292025 |
| hsa-miR-5701 | TXN | 7295 | HITS-CLIP | 27150721 |
| hsa-miR-450a-1-3p | IRAK3 | 11213 | HITS-CLIP//PAR-CLIP | 23446348\|21572407\|20371350\|23824327 |
| hsa-miR-561-5p | TXN | 7295 | HITS-CLIP | 23313552\|24038734\|24906430\|26061048\|27150721 |
| hsa-miR-1304-3p | NFATC2 | 4773 | HITS-CLIP | 23824327\|27418678 |
| hsa-miR-3184-3p | TXN | 7295 | PAR-CLIP | 27292025 |
| hsa-miR-548o-5p | TXN | 7295 | PAR-CLIP | 37986224 |
| hsa-miR-548am-5p | TXN | 7295 | PAR-CLIP | 37986224 |
| hsa-miR-1233-5p | IRAK3 | 11213 | HITS-CLIP//PAR-CLIP | 23446348\|21572407\|20371350\|23824327 |
| hsa-miR-5787 | IRAK3 | 11213 | HITS-CLIP | 23824327 |
| hsa-miR-6126 | IRAK3 | 11213 | HITS-CLIP | 23824327 |
| hsa-miR-6503-3p | GSTO1 | 9446 | PAR-CLIP | 27292025 |
| hsa-miR-6504-3p | NFATC2 | 4773 | HITS-CLIP | 23824327 |
| hsa-miR-6513-5p | IRAK3 | 11213 | HITS-CLIP//PAR-CLIP | 23446348\|21572407\|20371350\|23824327 |
| hsa-miR-370-5p | IRAK3 | 11213 | HITS-CLIP | 24389009 |
| hsa-miR-370-5p | S100A9 | 6280 | HITS-CLIP | 24389009 |
| hsa-miR-383-3p | IRAK3 | 11213 | HITS-CLIP//PAR-CLIP | 23446348\|23824327 |
| hsa-miR-605-3p | IRAK3 | 11213 | HITS-CLIP | 23824327 |
| hsa-miR-889-5p | TXN | 7295 | PAR-CLIP | 22100165 |
| hsa-miR-887-5p | IRAK3 | 11213 | HITS-CLIP//PAR-CLIP | 23446348\|21572407\|20371350\|23824327 |
| hsa-miR-6751-5p | GSTO1 | 9446 | PAR-CLIP | 27292025 |
| hsa-miR-6752-5p | GSTO1 | 9446 | PAR-CLIP | 27292025 |
| hsa-miR-6760-3p | NFATC2 | 4773 | HITS-CLIP | 23824327\|27418678 |
| hsa-miR-6779-5p | IRAK3 | 11213 | HITS-CLIP//PAR-CLIP | 23446348\|21572407\|20371350\|23824327 |
| hsa-miR-6780a-5p | IRAK3 | 11213 | HITS-CLIP//PAR-CLIP | 23446348\|21572407\|20371350\|23824327 |
| hsa-miR-6785-5p | IRAK3 | 11213 | HITS-CLIP//PAR-CLIP | 23446348\|21572407\|20371350\|23824327 |
| hsa-miR-6788-5p | IRAK3 | 11213 | HITS-CLIP//PAR-CLIP | 23446348\|21572407\|20371350\|23824327 |
| hsa-miR-6799-5p | IRAK3 | 11213 | HITS-CLIP//PAR-CLIP | 23446348\|21572407\|20371350\|23824327 |
| hsa-miR-6803-5p | GSTO1 | 9446 | PAR-CLIP | 27292025 |
| hsa-miR-6808-5p | IRAK3 | 11213 | HITS-CLIP//PAR-CLIP | 23446348\|21572407\|20371350\|23824327 |
| hsa-miR-6835-5p | GSTO1 | 9446 | PAR-CLIP | 27292025 |
| hsa-miR-6842-5p | GSTO1 | 9446 | PAR-CLIP | 27292025 |
| hsa-miR-6882-5p | TXN | 7295 | PAR-CLIP | 22100165 |
| hsa-miR-6883-5p | IRAK3 | 11213 | HITS-CLIP//PAR-CLIP | 23446348\|21572407\|20371350\|23824327 |
| hsa-miR-6893-5p | IRAK3 | 11213 | HITS-CLIP//PAR-CLIP | 23446348\|21572407\|20371350\|23824327 |
| hsa-miR-7106-5p | IRAK3 | 11213 | HITS-CLIP//PAR-CLIP | 23446348\|21572407\|20371350\|23824327 |
| hsa-miR-7109-5p | GSTO1 | 9446 | PAR-CLIP | 27292025 |
| hsa-miR-7110-5p | GSTO1 | 9446 | PAR-CLIP | 27292025 |
| hsa-miR-7157-5p | GSTO1 | 9446 | PAR-CLIP | 27292025 |
| hsa-miR-7157-5p | NFATC2 | 4773 | PAR-CLIP | 22100165 |
| hsa-miR-1273h-5p | IRAK3 | 11213 | HITS-CLIP//PAR-CLIP | 23446348\|21572407\|20371350\|23824327 |
| hsa-miR-450a-2-3p | GSTO1 | 9446 | PAR-CLIP | 27292025 |
| hsa-miR-7977 | IRAK3 | 11213 | HITS-CLIP | 23824327 |
| hsa-miR-8485 | NFATC2 | 4773 | HITS-CLIP | 23824327 |
| hsa-miR-196a | S100A9 | 6280 | other | 19342367 |
| hsa-miR-12135 | TXN | 7295 | HITS-CLIP | 26061048 |
| hsa-miR-320a-3p | IRAK3 | 11213 | HITS-CLIP | 24389009 |
| hsa-miR-320a-3p | NFATC2 | 4773 | HITS-CLIP//PAR-CLIP | 26061048\|26701625\|27292025 |
| hsa-miR-320a-3p | TXN | 7295 | HITS-CLIP | 24389009 |
| hsa-miR-9985 | IRAK3 | 11213 | HITS-CLIP | 24389009 |
| hsa-miR-9985 | S100A9 | 6280 | HITS-CLIP | 27150721 |
